# The role of social-psychological factors in the adoption of push-pull technology by small-scale farmers in East Africa: Application of the theory of planned behavior

**DOI:** 10.1016/j.heliyon.2024.e41449

**Published:** 2024-12-26

**Authors:** Denis Waiswa, Beatrice Wambui Muriithi, Alice W. Murage, Dave Mwangi Ireri, Fredah Maina, Frank Chidawanyika, Fahri Yavuz

**Affiliations:** a International Centre of Insect Physiology and Ecology (ICIPE), P.O. Box 30772-00100, Nairobi, Kenya; b Department of Agricultural Economics, Faculty of Agriculture, Atatürk University, Yakutiye, Erzurum, 25240, Türkiye; c Kenya Agricultural and Livestock Research Organization (KALRO), P.O. Box, 57811, 00200, Nairobi, Kenya; d Chuka University, P.O. Box 109, 60400, Chuka, Kenya; e Department of Zoology and Entomology, University of the Free State, P.O. Box 339, Bloemfontein, 9300, South Africa

**Keywords:** Adoption intention, Agricultural innovations, Agroecology, Artificial neural network, Innovation readiness, PLS-SEM

## Abstract

Push-pull technology (PPT) continues to gain relevance among smallholder farmers across the East African region in managing the constraints affecting cereal crop yields including stemborers, fall armyworm, striga weed, and low soil fertility. While previous research has emphasized the significance of socioeconomic factors in explaining farmers' decisions to adopt PPT, the social-psychological factors that influence farmers' adoption intentions have not been extensively studied. Therefore, this study investigated the influence of social-psychological factors on the intention to adopt or increase the land area under PPT based on the theory of planned behavior (TPB). Moreover, this study extends the applicability of the TPB by incorporating an additional construct that addresses practical limitations within adoption decision-making, and providing evidence of how TPB constructs function differently across distinct agricultural contexts. Nine hundred and seventy-one (971) cereal growers comprising PPT users and non-users were interviewed in Kenya, Uganda, Tanzania, and Rwanda using a structured questionnaire. Utilizing the Partial Least Square-Structural Equation Modelling (PLS-SEM) and Artificial Neural Network (ANN), this study revealed a significant positive influence of TPB conceptual elements attitudes, subjective norms, and perceived behavioral control on farmers’ intentions to adopt or expand the land area under PPT. Additionally, the study found that the added construct “perceived limitations” negatively impacts these intentions. The results indicated significant variations in the impact of TPB constructs on the intentions to adopt and expand land under PPT across the studied countries. These insights underscore the need for tailored, context-sensitive interventions that integrate behavioral, social, and practical considerations. Additionally, understanding the country-specific determinants allows for more targeted policy measures, extension services, and support programs that can enhance the adoption and effectiveness of PPT, ultimately improving agricultural outcomes and livelihoods of farmers in this region.

## Highlights

• PPT helps East African farmers manage cereal pests, weeds, and low soil fertility.
• Study explores social-psychological factors in PPT adoption, under-researched till now.
• Utilized TPB, PLS-SEM, and ANN to analyze adoption intentions among 971 cereal growers.
• Significant positive influence of attitudes, norms, and control; negative of perceived limitations.
• Country-specific TPB impacts suggest tailored interventions for improved PPT adoption.

## 1 Introduction

Agricultural productivity faces numerous challenges, with pest infestations, invasive weeds, and soil degradation as prominent factors affecting crop yields, food security, and rural livelihoods. In Sub-Saharan Africa (SSA), these challenges are particularly pressing, given the region's high dependence on rain-fed agriculture, limited access to agricultural inputs, and socio-economic constraints faced by smallholder farmers [1,2]. Here, cereal crops such as maize, sorghum, and millet are staple foods essential for food security. However, crop productivity is severely hindered by pests like stemborers, fall armyworm, and parasitic weeds such as *Striga* spp [1,3,4]. Stemborers cause yield losses as high as 80 % of the potential grain output [2,5]. Striga weed causes yield losses between 30 and 100 %, making the cooccurrence of the pests and the parasitic weeds a major societal concern [1]. The fall armyworm, due to its polyphagous nature, also causes extensive damage to many other crops including strategic cereals such as sorghum, millet, and rice. For maize, a recent study conducted on 343 smallholder farmers in Ethiopia and Kenya, revealed a 32 % average crop damage in Ethiopia and 47.3 % in Kenya, resulting in reduction in yield of approximately 0.8–1 ton per hectare [6]. In another study involving 791 smallholder maize plots in two districts of Eastern Zimbabwe, Baudron et al. [7] estimated an 11.6 % decrease in yield caused by the fall armyworm.

These losses, exacerbated by the ongoing effects of climate change, coupled with limited soil fertility, primarily impact smallholder farmers who rely heavily on these crops for their livelihood and nutrition, leading to significant levels of poverty, food insecurity, and malnutrition [[1], [2], [3],^8^]. Across various countries, farmers employ multiple approaches to combat these issues, ranging from chemical pesticides to integrated pest management techniques [1]. However, these solutions often come with trade-offs, such as environmental degradation, increased production costs, and health risks for farmers and consumers alike [1,2]. Moreover, given the constraints in access to resources like pesticides and fertilizers, sustainable, low-cost agricultural technologies are crucial to address these yield gaps. Push-Pull Technology (PPT), developed by the International Centre of Insect Physiology and Ecology (*icipe*) and partners in Kenya in the 1990s, is one such solution [2,4].

PPT provides an integrated, environmentally friendly approach that helps smallholder farmers manage pests and weeds while improving soil health, making it a viable option for SSA's unique challenges [[2], [3], [4]]. By simultaneously managing foliar pests such as stemborers and fall armyworm, parasitic weeds like *Striga* spp., and poor soil conditions, PPT offers a comprehensive approach to enhancing agricultural productivity in the region [5,9,10]. The technology involves intercropping desmodium (e.g., *Desmodium uncinatum* or *Desmodium intortum (Mill. Urb.)* between rows of cereals such as maize and sorghum to repel pests through airborne semiochemicals whilst the root exudates suppresses striga weed through suicidal germination [11]. The plot is also enclosed by a border crop, either Napier grass *(Pennisetum purpureum Schumach)* or Brachiaria spp., which lure pests away from the crop but without permitting complete larval development, serving as a dead-end trap [3,12,13]. PPT has demonstrated effectiveness in controlling these pests and weeds, thereby reducing losses attributable to these constraints [1,5]. For instance, PPT reduces up to 86.7 % of the damage caused by fall armyworm and other pests [3] with substantial yield and economic gains compared to monocultural cropping [1,5,14,15]. Additionally, it plays a vital role in climate change adaptation and mitigation efforts [16]. Moreover, the technology is environmentally benign as it has minimal impact on non-target organisms [4].

Despite these substantial benefits of PPT, its adoption remains relatively low. This low adoption continues to prevail, despite the considerable dissemination efforts put forward by development and extension agencies and research institutions, to promote the technology's adoption. This suggests the crucial need for implementing more targeted strategies to efficiently meet the needs of small-scale farmers in the region. In the realm of agricultural technology adoption, a substantial body of literature has emerged, emphasizing the significance of socioeconomic factors [[17], [18], [19], [20], [21]]. This literature recognizes the crucial role played by socioeconomic variables in shaping individuals' choices and actions regarding technology adoption. Additionally, numerous studies examining the drivers of PPT adoption and expansion of the land area have been conducted since its introduction in the late 1990s to date. These studies also emphasize the role of socioeconomic factors in determining the adoption of this technology. These factors include farmers' age, education, gender, marital status, land ownership, livestock ownership, dissemination pathways, access to input markets, access to extension services, membership of farmer groups, and credit availability [14,[22], [23], [24]].

Although these studies present important insights into the drivers of PPT adoption, there has been limited examination of the social-psychological factors that could potentially influence farmer's PPT adoption decisions. Meanwhile, recent research has placed significant emphasis on analyzing the attitudes, norms, and perceptions that are integral to the decision-making processes of farmers regarding specific agricultural technologies [25]. This emphasis stems from recognizing the profound impact these factors have on shaping farmers' adoption decisions. Understanding the impact of social-psychological factors is crucial for several reasons: i) viewing adoption as an ongoing decision-making process for farmers, where they continuously assess, adapt, and decide when to implement technologies [26]. ii) The commonly considered socioeconomic factors may be insignificant or have less impact on adoption [26]. Lastly, farmers' perceptions not only determine their decisions to adopt a technology, but also their decisions to encourage fellow farmers to adopt through peer-to-peer learning [10,27].

This study addresses the gap in the PPT adoption literature by exploring the social-psychological dimensions that shape farmers' adoption decisions. Specifically, this study examines how attitudes, perceived behavioral control, and subjective norms impact farmers' intentions. It focuses on non-users' intentions to adopt PPT and current users' intentions to expand the land area under PPT. By examining these factors, this study aims to shed light on farmers' decision behavior, a facet that is often overlooked in research focusing solely on socioeconomic factors. Incorporating these factors will yield benefits in two key aspects. Firstly, it will distinguish our study from previous studies, particularly those examining PPT adoption. Secondly, it will provide an additional benefit by elucidating farmers' perceptions that form the basis of their norms, attitudes, perceived control, and intentions to adopt PPT. This study utilizes Ajzen's (1991) Theory of Planned Behavior (TPB) as its conceptual framework.

Here, the TPB offers a valuable framework for understanding these factors, as it focuses on attitudes, perceived behavioral control, and subjective norms that shape individuals' intentions and behaviors [28]. Unlike other behavioral theories such as the Diffusion of Innovations theory, which emphasizes the spread and characteristics of innovations [29], or the Technology Acceptance Model (TAM), which primarily focuses on perceived usefulness and ease of use in technology uptake [30], TPB allows us to capture the complex, socially influenced motivations behind farmers' intentions to adopt PPT [31,32]. This study extends the TPB framework by introducing a new construct, “Perceived Limitations,” which accounts for farmers' perceived constraints, such as labor intensity and costs associated with PPT. This expanded model provides a more holistic view of farmers' behavioral intentions, addressing both motivational and contextual challenges specific to PPT adoption in East Africa. Unlike prior studies, this research not only seeks to understand the intentions of non-users to adopt PPT but also investigates current users’ decisions to expand their land area under PPT, thereby adding a dual focus that broadens our understanding of adoption pathways. Moreover, by conducting a cross-country analysis across Kenya, Uganda, Tanzania, and Rwanda, this study offers insights into both country-specific and broader regional patterns in PPT adoption behavior. These findings provide policymakers with a nuanced view of how internal beliefs and external social pressures drive adoption, enabling the development of more targeted and effective strategies that integrate socioeconomic and social-psychological factors to enhance the sustainability of PPT in East Africa.

## 2 The theory of planned behavior (TPB) and agricultural technology adoption

The TPB, proposed by Ajzen [28], suggests that individuals' behavior is influenced by their intentions, which are in turn shaped by their attitude toward the behavior, perceived behavioral control, and subjective norms [[32], [33], [34]]. Intention refers to an individual's motivation to put effort into carrying out a behavior [35]. The stronger an individual's intention to perform a behavior, the greater the likelihood of its actualization [33,34]. Attitude is defined by a positive or negative evaluation of engaging in a particular behavior. This construct is composed of behavioral beliefs and evaluation of behavioral outcomes. Therefore, farmers' intentions to adopt PPT or expand the land area dedicated to this technology will rise if they believe that utilizing this practice is advantageous and will yield positive outcomes for them.

Subjective norms pertain to an individual's perception of the social influence or expectations that encourage or discourage the performance of a particular behavior. This construct also highlights that individuals are influenced by societal figures such as family members, religious leaders, extension agents, and neighbors. Therefore, if farmers perceive that individuals whose opinions they respect endorse a particular behavior, their own intention to engage in that behavior is likely to increase [35]. Finally, perceived behavioral control encompasses a person's perception of their ability to control the behavior, which can directly or indirectly affect their intentions and subsequent behavior. This pertains to the ease or difficulty of carrying out a specific behavior and the availability of supportive conditions [35]. Consequently, farmers' intentions to adopt PPT are expected to rise as their perceived control over executing this behavior increases.

By aggregating these constructs, a positive or negative inclination towards carrying out a particular behavior can be attained. To foster behavioral change, policymakers, research institutes, and other stakeholders must adeptly translate these drivers and barriers into actionable plans [32]. Nevertheless, researchers have identified limitations in the TPB's three constructs, suggesting that they may not comprehensively address all factors influencing intention. Additionally, scholars argue that the TPB could benefit from the incorporation of additional constructs i.e., if another influential factor can account for a significant portion of the variation in intention or behavior, it should be considered for integration into the theory [31,36]. This is essential to bolster the model's explanatory capacity and offer a more holistic understanding of the TPB constructs. This has prompted several authors including Lalani et al. [26], Bagheri et al. [33], Hyland et al. [32], Adnan et al. [37], and Ataei et al. [31], to expand upon these constructs by introducing additional variables.

**Fig. 1:**
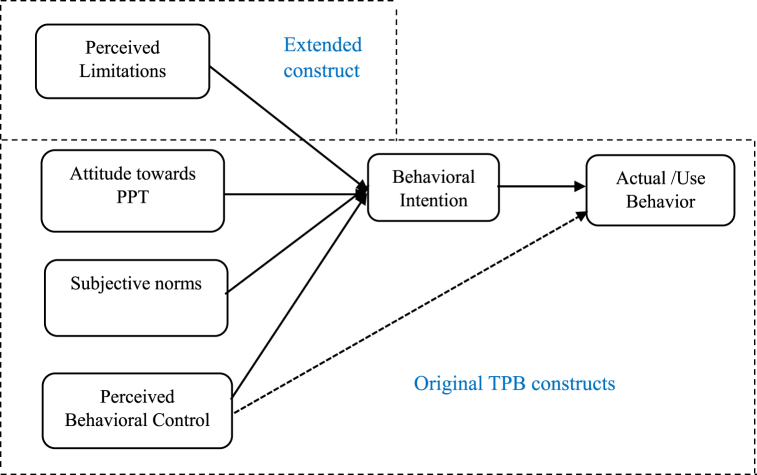
The conceptual framework of the TPB, Adapted from Despotović, et al. [40].

In this regard, we extended the original TPB framework by introducing an additional construct “Perceived Limitations” as illustrated in Fig. 1. Although PPT offers substantial benefits, farmers have reported certain limitations that may impact their decision to adopt or expand the use of this technology. Specifically, three primary limitations have been reported in literature: (1) higher labor requirements, particularly during the initial years of establishment [14,24,38]; (2) restricted flexibility for crop rotation [38]; and (3) limited direct utility of companion plants for human consumption [38], aside from cereals and the recently introduced vegetable-integrated PPT. Therefore, the "Perceived Limitations" construct was designed to capture farmers’ views on these constraints, as each aspect has implications for the adoption process.

First, the increased labor requirement is often a critical consideration for smallholder farmers who may have limited access to labor or face high costs associated with hiring additional help [14,39]. This labor-intensive aspect of PPT, especially in the initial years of adoption, can deter adoption for resource-constrained households. Second, the lack of flexibility in crop rotation means that PPT may not align well with farmers' long-term rotational practices or cropping plans, potentially limiting its perceived suitability for diverse farming systems. Finally, since the companion plants used in PPT (such as desmodium) are generally not consumed by humans, they may be viewed as less beneficial compared to food crops, especially in resource-scarce settings where each crop must ideally serve multiple purposes [38].

To operationalize the "Perceived Limitations" construct, we included survey variables that measured farmers’ perceptions of the labor, time, and cost requirements associated with PPT as presented in Table 1. These variables were structured to assess the degree to which these perceived limitations might deter farmers from initially adopting PPT or expanding their existing PPT acreage. By analyzing these responses, we aimed to identify whether farmers who perceive higher limitations in terms of labor demands, costs, or rotational inflexibility exhibit lower intentions to adopt or expand PPT. The findings from this construct provide critical insights into the potential barriers that could be addressed through tailored interventions, such as broader crop integration strategies, to make PPT more attractive and feasible for a wider range of smallholder farmers.

**Table 1:** Variables with their references.

Construct	Variable	Description	References
Attitude	ATT1	PPT can help increase production or yields on the farm	Chepchirchir, et al. [1],Kassie, et al. [2],Ndayisaba, et al. [4],Khan, et al. [5],Amudavi, et al. [9],Murage, et al. [14],Ndayisaba, et al. [27],Diiro, et al. [39]
	ATT2	PPT can help increase overall farm profits	
	ATT3	PPT can help ensure availability of enough fodder for livestock	
	ATT4	PPT can help reduce soil erosion	
	ATT5	PPT can help reduce striga weed infestation	
	ATT6	PPT can help control stemborers	
	ATT7	PPT can help control fall armyworm	
	ATT8	PPT can improve soil fertility, soil health, and water retention	
	ATT9	PPT can help to rehabilitate the land	
	ATT10	Using PPT reduces the need to use chemical/synthetic fertilizers	
	ATT11	Using PPT reduces the need to use pesticides (insecticides)	
	ATT12	Using PPT reduces the need to use herbicides	
	ATT13	PPT can help reduce labor and costs for weeding	
	ATT14	PPT can help improve resilience of crops to climate change	
	ATT15	I like trying new agricultural technologies or practices	
Perceived Limitations	PL1	PPT requires additional labor	Hyland, et al. [32],Diiro, et al. [39]
	PL2	PPT can be time consuming	
	PL3	PPT imposes extra costs	
	PL4	PPT cannot be practiced on large piece of land	
	PL5	PPT does not allow for crop rotation	
Perceived Behavioral Control	PBC1	I have all the information I need to implement PPT	Ataei, et al. [31],Bagheri, et al. [33],Rezaei, et al. [36],Tama, et al. [43],Ong, et al. [47],Huang [48]
	PBC2	I have a good understanding of how PPT works	
	PBC3	I have resources to implement PPT	
	PBC4	Initial costs of PPT are reasonable considering that overall costs reduce in the following seasons and the increase in productivity output	
	PBC5	PPT input markets such as seeds are in the vicinity and inputs are easily available to me	
	PBC6	Cultivating crops without using synthetic chemicals (fertilizers, herbicides, and pesticides) is achievable for me	
	PBC7	The implementation of PPT is reasonably uncomplicated, and I can easily implement the technology on my land	
	PBC8	I am confident enough in my ability and competency to implement PPT	
Subjective Norms	SN1	Farmers who use PPT harvest more products	Hyland, et al. [32],Bagheri, et al. [33],Li, et al. [34],Despotović, et al. [40]
	SN2	Farmers in my surroundings use PPT to prevent striga weed, stemborers, and fall armyworm	
	SN3	A member of my family would recommend using PPT	
	SN4	Other farmers would recommend using PPT.	
	SN5	My farmer group or cooperative would recommend using PPT.	
	SN6	My extension advisor would recommend using PPT.	
	SN7	Conditions on-farm (would) allow for the implementation of PPT	
Intention^1^	INT1	My main goal in farming is to maximize production	Ataei, et al. [31],Bagheri, et al. [33],Tama, et al. [43],Ong, et al. [47]
	INT2	I intend to reduce pesticide applications in my crops	
	INT3	I intend to reduce herbicide applications in my crops	
	INT4	I plan to adopt PPT this year (next planting season) or next year	
	INT5	I plan to adopt PPT in the next two years	
	INT6	I plan to adopt PPT sometime in the future	
	INT7	I intend to encourage other farmers to adopt PPT	
	INT8	I intend to adopt PPT or increase the farm area under PPT	
Actual Behavior	ABEH	Is your household currently using PPT? Yes = 1, No = 0	

The TPB has been widely adopted to study farmers' decisions in adopting agricultural technologies due to its ability to offer a valid model for explaining their intentions and its role in identifying knowledge gaps in technology dissemination [31,32]. While explaining 80 % of the variation in farmers’ intentions, the TPB provided a valid model for explaining farmers' intentions to adopt Conservation Agriculture [26]. In other studies, the TPB has been utilized to explore farmers' intentions regarding various technologies aimed at enhancing agricultural productivity. These include management of nutrients [35], pressurized irrigation [41], integrated crop management [42], Integrated Pest Management (IPM) [40], Conservation Agriculture (CA) [25,26,43], and Sustainable Agricultural Practices (SAPs) [37,44].

These studies demonstrate how the three components of TPB influence farmers' intentions to adopt agricultural technologies, how farmers' intentions shape their actual behavior, and the potential interrelationships among the TPB constructs. In most studies, these three constructs have positively and significantly influenced farmers' adoption intentions. These studies further illustrate the differences in the magnitude of impact of the three TPB constructs. For example Bagheri et al. [42] and Wang et al. [45] reported that subjective norms had the strongest impact on farmers' adoption intentions. Lalani et al. [26] unveiled that attitude exerted the most influential effect on their adoption intentions. Finally, Daxini et al. [35] and Daxini et al. [46] revealed that perceived behavioral control had the greatest influence on farmers’ intentions.

The lack of consensus regarding the impact of these constructs on adoption intentions emphasizes the importance of analyzing their influence on specific technologies. Moreover, Ajzen [28] asserted that the impact of TPB constructs varies based on the behavior and context being examined. This necessity is particularly pronounced for PPT, as no study has examined the role of the TPB components in farmers' intentions to adopt the technology, their subsequent behavior, and the potential interrelationships between these constructs. Furthermore, the aforementioned studies exclusively centered on examining the impact of TPB constructs on the intentions to adopt agricultural technologies. In contrast, our study employs three distinct models.

The first model evaluates the impact of TPB constructs on the adoption intentions of farmers regarding PPT, specifically among those who have not yet adopted the technology (here after referred to as "non-users"), following the approach utilized in prior research. The second model assesses the influence of TPB constructs on the intentions of farmers to expand the land area allocated to PPT, this time targeting farmers who are already utilizing the technology (here after referred to as "users"). The third model incorporates both users and non-users of PPT within the same framework (here after referred to as the "pooled sample"), enabling a comprehensive examination of the influence of these constructs on farmers' intentions, as well as their subsequent effects on actual adoption behavior. Additionally, apart from estimating a comprehensive model using aggregated data from the four countries (Kenya, Uganda, Tanzania, and Rwanda), we conduct separate model estimations for each country. This approach allows us to discern any variations in the impact of TPB constructs on adoption intentions across different national contexts.

### 2.1 Hypotheses

This study examines the impact of attitude, perceived behavioral control, subjective norms, and perceived limitations, on PPT adoption intentions and the actual adoption behavior. The items for each construct were sourced from existing literature and tailored to the context of PPT adoption. These variables were examined using observed statements, rated on a five-point Likert scale that ranged from “strongly disagree = 1” to “strongly agree = 5” and are presented in Table 1. Drawing from previous studies, this study hypothesizes that:

H1 Farmers' attitudes, subjective norms, and perceived behavioral control positively impact their adoption intentions among non-users and their expansion of land area allocated to the technology among current users.

H2 Farmers' perceptions of PPT limitations negatively impact their adoption intentions among non-users and their expansion of land area allocated to PPT among current users.

H3 Farmers' behavioral intentions and perceived behavioral control positively impact their actual PPT adoption behavior.

## 3 Material and methods

### 3.1 Description of the study area, sample selection, and data collection

This study is part of the UPSCALE project, *"Upscaling the Benefits of Push-Pull Technology for Sustainable Agricultural Intensification in East Africa."* More information about the project is available on the website (https://upscale-h2020.eu/about-us/). The project is being implemented in Kenya, Ethiopia, Rwanda, Uganda, and Tanzania. Data collection for the project occurs at three key points: the baseline survey in 2021 at the project's inception, a midline survey in 2023, and an endline survey planned for 2025. During the baseline survey, 1556 farmers from the five project countries were interviewed. The survey utilized a longitudinal survey design to collect data from both PPT and non-PPT farmers. Each country targeted 300 cereal-growing households from specific project sites, including: Kisumu, Vihiga, Siaya, and Homabay counties in Kenya, North Shewa, Oromia Special zone, and South Wollo in Amhara in Ethiopia, Butiama, Bunda, Rorya, and Tarime districts in Mara region in Tanzania, Nyagihanga Sector in Gatsibo district in Rwanda, and Iganga, Kamuli, and Namutumba districts in Uganda.

A multi-stage sampling approach was used to select administrative units and respondents. Initially, regions with previous PPT dissemination were purposefully selected. Then, administrative units within these regions were chosen based on past PPT adoption efforts. Extension officers and country project partners provided a census of cereal growers, both PPT and non-PPT. Respondents were randomly selected using probability proportional to size (PPS) sampling, while non-PPT households were chosen through systematic random sampling. Data was collected by trained enumerators familiar with local languages, using the Open Data Kit (ODK) tool to collect both quantitative and qualitative data via a closed-ended questionnaire. The questionnaire was co-designed by the UPSCALE consortium partners, led by the socioeconomics team from the Kenya Agricultural and Livestock Research Organization (KALRO) and the International Centre of Insect Physiology and Ecology (*icipe*).

**Fig. 2:**
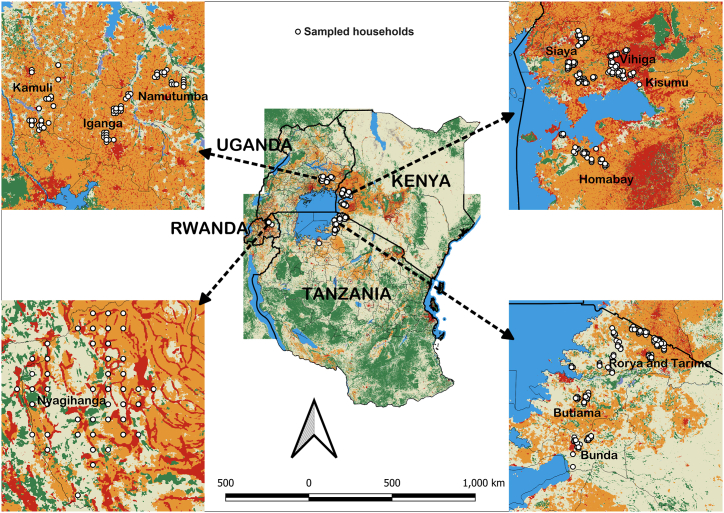
Distribution of the study areas in the four countries that were sampled for the midline survey.^2^

The midline survey, conducted from August to September 2023, followed up with the same farmers from the baseline, except in Ethiopia where civil unrest prevented data collection. A total of 1237 farmers were interviewed from study sites in Kenya, Rwanda, Tanzania, and Uganda (see Fig. 2). For this study's analysis, we utilized only data collected during the midline survey because the baseline questionnaire did not include questions related to the theory of planned behavior.

### 3.2 Statistical analysis

This study integrates both Partial Least Squares SEM (PLS-SEM) and Artificial Neural Network (ANN) methodologies to leverage their complementary strengths. PLS-SEM is employed primarily for hypothesis testing and to assess causal relationships among constructs, while ANN is applied as a supplementary tool to validate the findings from PLS-SEM and to capture potential nonlinear interactions that PLS-SEM cannot account for [49,50].

Data analysis and hypothesis testing were performed using the SmartPLS 4.0 statistical software [51]. PLS-SEM was selected for this study over covariance-based SEM (CB-SEM) due to its effectiveness in examining highly complex models consisting of multiple constructs, latent variables, and structural paths [25,45,52]. It also offers a more flexible approach compared to CB-SEM when it comes to assuming the distribution of variables and error terms i.e., multivariate normality in the dataset is not essential [45,52,53]. PLS-SEM also possesses the valuable capability of being applicable even to smaller sample sizes [53]. Moreover, PLS-SEM effectively identifies the direction and strength of linear relationships, validating the theoretical model by testing the hypothesized paths among the constructs derived from the TPB. We utilized bootstrapping using a sub-sample size of 5000 to assess the significance of the coefficients.

However, PLS-SEM has limitations when it comes to accommodating nonlinear relationships, which may restrict its capacity to detect more complex interactions among variables [53]. To address this, we implement ANN as an advanced analytic tool for deeper insights [49,50]. ANN can model both linear and nonlinear relationships, using its feedforward-backpropagation structure to iteratively learn and refine predictions based on the dataset [50,54]. While ANN does not test causal relationships directly, it can validate the relative importance of variables, which adds robustness to the findings derived from PLS-SEM. Moreover, ANN is important in feature selection by identifying the relative importance of each TPB construct (and perceived limitations) in predicting adoption and expansion intentions. This added layer of analysis is important for confirming the strength and significance of each predictor variable identified through PLS-SEM, reinforcing the validity of our findings across multiple analytical techniques.

Additionally, while PLS-SEM provides a structured, theory-driven framework to test causal relationships, ANN complements this by validating these relationships and uncovering hidden nonlinear patterns, resulting in a more robust and reliable analysis [50,54]. Moreover, ANNs identify critical factors by examining complex relationships between predictors and outcomes, without requiring multivariate assumptions like normality and homoscedasticity [53]. However, ANN's "black box" nature limits its utility for testing causal relationships, necessitating caution when interpreting results [50,53]. Therefore, the dual-method approach utilized in this study validates the consistency of TPB-related constructs, ultimately enhancing the reliability of insights and strengthening the study's conclusions, enhancing its policy implications. ANNs have been applied across various fields, including entrepreneurial intentions [55], consumer behavior [50], mobile health services [53], advertising strategies [49], and mobile wallet resistance [54].

## 4 Results and discussion

### 4.1 Socioeconomic characteristics of the sample

Out of the 1237 interviewed farmers, 971 farmers were aware of PPT and only these responded to the TPB statements, forming the basis of this study's analysis. Although not included in the analysis, we present some characteristics of the sampled farmers in Table 2. This is intended to offer a comprehensive demographic and socioeconomic profile of the surveyed sample, enriching the study's contextual understanding, and facilitating more nuanced interpretations of the findings. Among the 971 farmers aware of PPT, 31.6 % were from Rwanda, 24.2 % from Kenya, 22.8 % from Uganda, and 21.4 % from Tanzania. Regarding demographic characteristics, 79.9 % of households were led by males, while 20.1 % were led by females. The average age of household heads was 52.6 years. Specifically, 29.2 % were between 51 and 60 years old, 26.5 % were over 60, 25.1 % were between 41 and 50, 15.9 % were between 30 and 40, and 3.3 % were under 30. The average education years of household heads was 9.3 years. The majority (56.4 %) had 7–14 years of education, followed by 39.8 % with less than 7 years of education. The average household size was 6.8 members, with 65.5 % of households having 5 to 10 members, 23.4 % having fewer than 5 members, and 11.1 % having more than 10 members.

**Table 2:** Socioeconomic characteristics of the sample.

Variable	Category	Frequency	Percent
Country	Kenya	235	24.2
	Uganda	221	22.8
	Tanzania	208	21.4
	Rwanda	307	31.6
Gender of the household head	Female	195	20.1
	Male	776	79.9
Main source of income	Otherwise	112	11.5
	Agriculture	859	88.5
Age of the household head	<30	32	3.3
	30–40	154	15.9
	41–50	244	25.1
	51–60	284	29.2
	>60	257	26.5
Household head's years of education	<7	386	39.8
	7–14	548	56.4
	15–17	34	3.5
	18–20	2	0.2
	>20	1	0.1
Household members	<5	227	23.4
	5–10	636	65.5
	>10	108	11.1
Land owned by the household (acres)	<5	799	82.3
	5–20	150	15.4
	21–40	6	0.6
	>100	16	1.6
Status of adoption	No	496	51.1
	Yes	475	48.9

Regarding economic activity, 88.5 % of the households surveyed practiced agriculture as their primary livelihood. On average, households owned 2.93 acres of land. Specifically, 82.3 % owned less than 5 acres, 15.4 % owned between 5 and 20 acres, and the rest owned above 20 acres. This aligns with existing literature, which indicates that agricultural production in Sub-Saharan Africa (SSA) is predominantly conducted on farms smaller than 2 ha [56], accounting for about 75 % of the region's agricultural output [57]. Regarding PPT adoption, 48.9 % of the farmers aware of the technology were users, while 51.1 % were non-users.

### 4.2 Common method bias

To evaluate the possible impact of common method bias (CMB) on our variables (those presented in Table 1), we conducted a Harman single-factor analysis, a widely used technique in academic research [55]. This test is necessary to ensure that the results are not unduly influenced by the measurement method, thereby validating our findings [58]. The results indicate that a single-factor analysis accounted for 23.7 %, 31.7 %, and 35.6 % of the variance among PPT users, non-users, and the pooled sample, respectively, which are lower than the 50 % threshold commonly accepted as representing no common method bias.

### 4.3 Reliability and validity of the model

In SEM, it is common practice to assess the model fit before conducting hypothesis testing. This step ensures the accuracy of the findings [31]. The measurement model assessment was conducted according to various key criteria, including indicator and convergent reliability, discriminant validity, and internal consistency. Reliability pertains to the internal consistency of the various indicators employed for measuring each construct [35]. To evaluate the indicator reliability, we examined the factor loadings, ensuring that most of those used in the analysis exceeded 0.7 as presented in Table 3. Additionally, the reliability of intention, attitude, perceived limitations, perceived behavioral control, and subjective norm statements were evaluated using Cronbach's alpha and composite reliability values. These values were above the minimum acceptable threshold of 0.7, indicating high reliability across all estimated models (Table A.1 in the appendix) [59].

**Table 3:** Characteristics of the variables utilized in the analysis.

Variable	PPT users				Non-users				Pooled sample			
	Mean	SD	M	OL	Mean	SD	M	OL	Mean	SD	M	OL
ABEH									0.489	0.500		1.000
ATT1	4.505	0.630	4.338		3.895	0.945	3.802	0.803	4.195	0.861	4.064	0.770
ATT2	4.411	0.670			3.842	0.900		0.811	4.120	0.844		0.774
ATT3	4.486	0.627			4.004	0.925		0.728	4.242	0.828		0.716
ATT4	4.331	0.673			3.864	0.892		0.749	4.092	0.827		0.716
ATT5	4.421	0.665		0.588	3.848	0.909		0.812	4.125	0.851		0.782
ATT6	4.442	0.698		0.715	3.941	0.893		0.815	4.186	0.841		0.777
ATT7	4.451	0.713		0.655	3.868	0.912		0.783	4.151	0.871		0.744
ATT8	4.375	0.631			3.828	0.853		0.802	4.096	0.800		0.780
ATT9	4.272	0.668			3.746	0.832		0.789	4.004	0.803		0.760
ATT10	4.229	0.717		0.723	3.688	0.835		0.797	3.953	0.824		0.760
ATT11	4.331	0.651		0.820	3.757	0.817		0.807	4.038	0.794		0.794
ATT12	4.269	0.704		0.769	3.740	0.812		0.823	3.999	0.805		0.778
ATT13	4.053	0.868			3.458	0.921		0.592	3.750	0.944		0.609
ATT14	4.131	0.714			3.592	0.817		0.663	3.856	0.813		0.668
ATT15	4.356	0.694			3.963	0.895			4.157	0.826		0.559
PL1	3.177	1.345	3.062	0.802	3.310	1.065	3.192	0.773	3.248	1.212	3.132	0.787
PL2	3.248	1.337		0.831	3.308	1.110			3.283	1.228		0.844
PL3	3.116	1.305		0.848	3.284	1.092		0.732	3.203	1.205		0.826
PL4	2.945	1.348		0.694	2.992	1.118		0.677	2.972	1.236		0.582
PL5	2.825	1.319		0.746	3.067	1.080		0.727	2.954	1.211		0.765
PBC1	3.958	0.942	3.826	0.728	3.047	1.121	3.028	0.755	3.493	1.131	3.420	0.792
PBC2	4.143	0.787		0.835	3.181	1.153		0.800	3.653	1.100		0.837
PBC3	3.577	1.141			2.505	1.091		0.540	3.034	1.238		0.695
PBC4	3.907	0.759		0.632	3.302	0.949		0.712	3.599	0.912		0.706
PBC5	3.179	1.240			2.505	1.115			2.831	1.226		0.536
PBC6	3.815	0.838			3.174	0.967		0.676	3.490	0.960		0.688
PBC7	3.943	0.773		0.651	3.274	0.938		0.822	3.601	0.924		0.768
PBC8	4.088	0.787		0.793	3.237	1.093		0.828	3.655	1.045		0.818
SN1	4.362	0.716	4.168	0.708	3.840	0.890	3.551	0.745	4.093	0.852	3.852	0.757
SN2	4.139	0.829			3.383	1.106		0.618	3.753	1.049		0.676
SN3	4.154	0.745		0.694	3.448	0.978		0.803	3.793	0.941		0.805
SN4	4.139	0.721		0.769	3.538	0.885		0.841	3.830	0.864		0.839
SN5	4.086	0.773		0.710	3.499	0.891		0.768	3.786	0.886		0.776
SN6	4.200	0.642		0.725	3.651	0.855		0.759	3.918	0.808		0.773
SN7	4.093	0.751		0.722	3.497	0.942		0.675	3.790	0.903		0.726
INT1	4.524	0.588	4.270		4.428	0.713	3.757		4.473	0.661	3.827	
INT2	4.257	0.700		0.792	3.949	0.859		0.529	4.100	0.799		0.728
INT3	4.196	0.727		0.788	3.959	0.761			4.075	0.752		0.652
INT4					3.402	1.126		0.573	3.406	1.126		
INT5					3.413	1.091		0.799	3.412	1.090		
INT6					3.547	1.095		0.745	3.547	1.094		
INT7	4.337	0.668		0.818	3.600	0.867		0.825	3.960	0.858		0.853
INT8	4.038	0.944		0.688					3.644	1.061		0.800

**Note:** M denotes the overall mean, SD denotes standard deviation, OL denotes the outer loadings.

Validity, on the other hand, pertains to the accuracy of the variables in measuring the targeted construct [35,55]. We employed two methods to assess validity: discriminant validity using Fornell and Larcker criteria [60], as well as the Heterotrait-Monotrait Ratio of Correlations (HTMT) [61]. In Table A.2, all constructs in the estimated models have values below the threshold values of 0.85 and 0.9, thereby demonstrating adherence to the HTMT ratio. The diagonal lines in Table A.3 in the appendix present the square roots of AVE values. These surpass the recommended value of 0.5 which conforms to the Fornell-Larker condition. These findings clearly indicate that the measurement models employed in this study have effectively demonstrated significant discriminant validity. This signifies that each construct within each model is distinct and can be differentiated from the others [31,35].

Convergent validity, which assesses the extent to which the observed variables align with each other, is evaluated using the average variance extracted for each construct in each model. An AVE value exceeding 0.5 is considered satisfactory [31]. In our study, all AVE scores for the estimated models are above 0.5, indicating satisfactory convergent validity (Table A.1). Additionally, we examined multicollinearity between the variables in the estimated models by calculating the variance inflation factors (Table A.4). VIF values below 5 were observed in the estimated models. This suggests that multicollinearity is not a concern in our models, ensuring the stability of our results [35,54].

### 4.4 Descriptive statistics of the measured items

The average scores of each construct in each model are displayed in Table 3. Notably, the intention of farmers to adopt PPT or expand the area of land allocated to PPT was high. The average scores for intentions stood at 4.27, 3.76, and 3.83 among users, non-users, and the pooled sample, respectively. This suggests a strong inclination towards PPT adoption among non-users and increasing the land area under PPT among current users of the technology. Farmers' high intention to adopt PPT stemmed from their intentions to reduce pesticide applications on their farms, their plans to adopt PPT in the upcoming planting season, within the next two years, or at some point in the future, and their efforts to encourage other farmers to adopt PPT. On the other hand, current users of PPT expressed high intentions to expand the area of their farms allocated to PPT usage, as well as a genuine motivation to reduce pesticide and herbicide applications on their farms, and encourage fellow farmers to adopt PPT.

Regarding attitudes, farmers expressed positive attitudes towards PPT. However, according to the average scores for the attitude statements, users of the technology expressed a higher positive attitude towards the benefits of the technology (4.34) compared to non-users (3.80). This suggests that non-users may either have limited understanding of the benefits of the technology or have lower confidence in PPT's benefits. Consequently, there may be a necessity for additional initiatives to effectively enhance farmers' awareness regarding the benefits associated with PPT. Farmers' positive attitudes towards PPT stemmed from their perception of it as a highly effective strategy for enhancing yields and overall profitability on their farms. They also viewed the technology as essential for ensuring a consistent supply of fodder for their livestock, mitigating soil erosion, managing the three major constraints (striga, fall armyworm, and stemborers), improving soil fertility, rehabilitating the land, reducing reliance on synthetic fertilizers, pesticides, and herbicides, reducing labor and weeding costs, and enhancing crop resilience in the face of climate change. PPT's ability to ensure fodder availability for livestock obtained the highest score of 4.0 among non-users of the technology. This suggests that emphasizing the crucial role played by PPT in ensuring a reliable supply of fodder for livestock, particularly among those who have not yet adopted the technology, could be an effective approach for promoting its adoption, alongside emphasizing the technology's ability to manage the target constraints.

The perceived behavioral control (PBC) construct was evaluated with a mean score of 3.83 and 3.03 among users and non-users, respectively. Even though this suggests an overall positive perception of farmers' abilities to implement PPT, the low scores, especially among the non-users suggest a limited access to the necessary inputs for PPT use. Moreover, when examining the various PBC statements, the possession of resources necessary for effectively implementing PPT and the accessibility to input markets were found to have the lowest mean scores among users, with scores of 3.58 and 3.18, respectively. Among non-users, both statements had a mean score of 2.51. Despite these low scores, farmers still expressed positive confidence in their own abilities and competencies to implement PPT, with mean scores of 4.09 and 3.24 among users and non-users, respectively. This suggests that the lack of resources and limited access to inputs pose primary challenges to the adoption of PPT. These findings are consistent with prior research by Cheruiyot et al. [3] and Librán-Embid et al. [38] which highlighted the limited access to companion plant seeds, especially Desmodium seeds, as barriers to the adoption of PPT. The PBC construct further consisted of statements regarding farmers’ understanding of how PPT works, their perception of the ease of implementation, the initial costs, possession of sufficient information, and their perception about cultivating crops without using synthetic chemicals (fertilizer, herbicides, and pesticides). The mean scores of these statements further highlight the significance of skills and knowledge in influencing perceived behavioral control regarding PPT.

The average score for subjective norms was found to be 4.17 and 3.55 among users and non-users, respectively, indicating a positive perception in both groups. Several factors contribute to the subjective norms construct, including their perception of the benefits experienced by other PPT-practicing farmers, and the influence of extension advisors, fellow farmers, and family members. Furthermore, the favorability of conditions on their farms, the impact of farmer groups or cooperatives, and the presence of PPT-practicing farmers in their surroundings also influence the subjective norms construct. The difference between the mean scores of users and non-users suggests that farmers may exhibit a greater propensity to embrace PPT if they perceive that their fellow farmers and reference groups highly value and endorse its adoption [25].

The average of perceived limitations statements was found to be 3.06 and 3.19 among users and non-users of the technology, respectively, indicating a moderate level of perceived limitations among the farmers in both groups. This construct was primarily associated with the farmers' understanding of the time, labor, cost, and land requirements of PPT. Additionally, PPT's limitation in allowing for crop rotation on the land further contributed to the perceived limitations. The low mean scores of the perceived limitations construct in the two groups compared to the other constructs particularly intentions to adopt or increase the land area under PPT demonstrate a high level of willingness to take up the technology despite its limitations.

### 4.5 Multi-group and path model analysis

The impact of TPB constructs on farmers' adoption intentions may differ across diverse national contexts, reflecting agricultural landscapes, socioeconomic dynamics, and policy frameworks unique to each country. For instance, in Rwanda, where smallholder farming predominates and government-led initiatives prioritize sustainable agricultural practices [62], TPB constructs may interact differently with adoption intentions compared to Kenya, where a mix of smallholder and commercial farming coexists, and governmental policies focus on market-oriented agricultural development [63]. Similarly, in Uganda, where subsistence farming is prevalent alongside emerging commercial ventures [64], and Tanzania, with its vast agricultural expanses and diverse agroecological zones [65], TPB constructs may manifest varying influences on farmers' adoption intentions. Understanding these nuanced differences within national contexts is crucial for tailoring interventions and policy measures that effectively promote the adoption of PPT. Therefore, we performed a PLS-SEM multi-group analysis to compare the outcomes across these four countries.

**Fig. 3:**
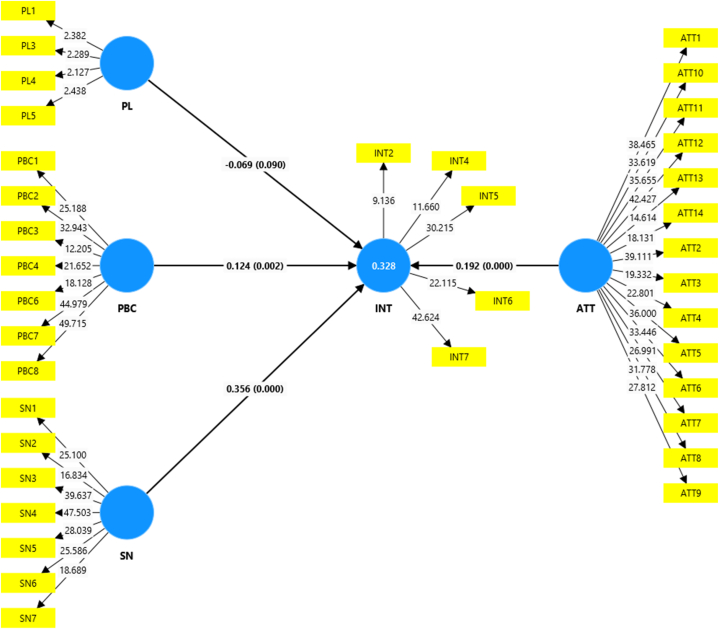
Structural model for non-users in the combined country model (figures in brackets are p-Values).

**Fig. 4:**
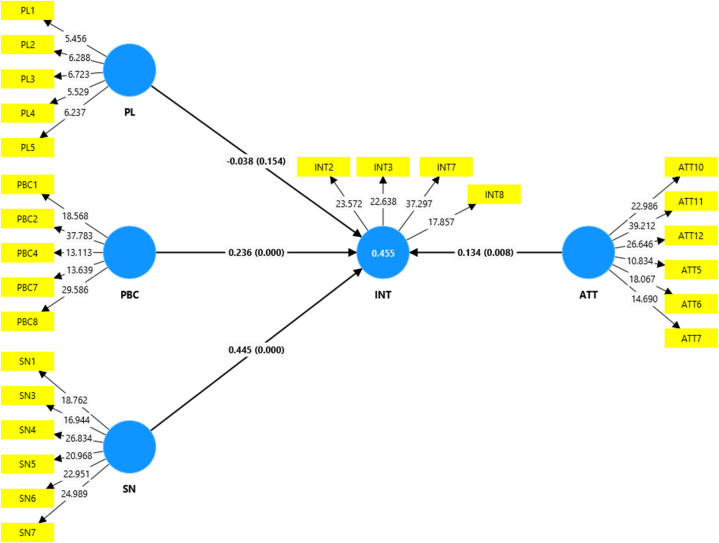
Structural model for PPT users in the combined country model (figures in brackets are p-Values).

**Fig. 5:**
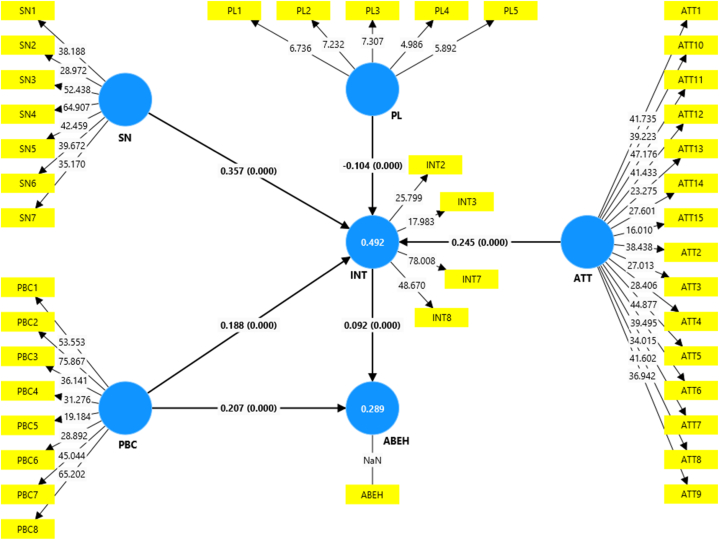
Structural model for the pooled sample in the combined country model (figures in brackets are p-Values).

The outcomes of the permutation test (conducted with 5000 permutations) reveal the findings of the PLS-SEM multi-group analysis, as depicted in Table A.5. These findings highlight statistically significant variations in the path coefficients specific to each group observed across the four countries, highlighting the importance of national contextual factors in shaping adoption behavior. We conducted a multi-group analysis utilizing the combined country models that incorporate aggregated data from all four countries. These are presented in Fig. 3, Fig. 4, Fig. 5 and the last column of Table 4. However, to ensure the models' reliability and validity, we re-estimate the models for each country separately. The results of each country's models can be found in Table 4 and Figures B.1 to B.12 in the appendix. In regard to the endogenous latent variable, adoption intention, the R-squared values for country-specific estimates surpass those derived from model estimations using the aggregated dataset, especially among both non-users and users of the technology. Therefore, the differentiation among countries provides a higher explanation power of the target constructs of the model and predictive accuracy, further bolstering the credibility of the country-specific estimates.

**Table 4:** Total effects of the model constructs on intention and actual adoption behavior.

	Kenya		Rwanda		Tanzania		Uganda		All countries combined	
	N = 105		N = 110		N = 138		N = 143		N = 496	
	O. sample (O)	T stat. (|O/SD|)	O. sample (O)	T stat. (|O/SD|)	O. sample (O)	T stat. (|O/SD|)	O. sample (O)	T stat. (|O/SD|)	O. sample (O)	T stat. (|O/SD|)
**Non-users**										
ATT - > INT	−0.088	0.628	0.344∗∗∗	3.317	0.522∗∗∗	6.096	0.029	0.269	0.192∗∗∗	3.323
PBC - > INT	0.286∗∗∗	3.131	0.126∗	1.594	−0.016	0.161	0.210∗∗∗	2.716	0.124∗∗∗	2.863
PL - > INT	−0.055	0.580	−0.217∗∗∗	3.187	0.116	1.107	−0.111∗	1.351	−0.069∗	1.340
SN - > INT	0.406∗∗∗	4.129	0.310∗∗∗	2.819	0.095	0.996	0.523∗∗∗	4.519	0.356∗∗∗	6.013
R2	0.273		0.568		0.327		0.428		0.328	
Q2	0.179		0.496		0.252		0.381		0.313	
**Users**	N = 130		N = 197		N = 70		N = 78		N = 475	
ATT - > INT	0.238∗∗∗	3.390	0.069	0.683	0.375∗∗∗	3.796	0.342∗∗∗	3.213	0.134∗∗∗	2.401
PBC - > INT	0.353∗∗∗	4.921	0.130	1.046	0.245∗∗∗	2.432	0.118∗	1.376	0.236∗∗∗	4.354
PL - > INT	−0.053	0.834	0.105∗	1.478	−0.401∗∗∗	4.467	−0.102	0.988	−0.038	1.019
SN - > INT	0.340∗∗∗	4.288	0.603∗∗∗	4.778	0.074	0.598	0.398∗∗∗	3.271	0.445∗∗∗	5.804
R2	0.621		0.504		0.531		0.526		0.455	
Q2	0.564		0.425		0.354		0.394		0.426	
**Pooled sample**	N = 235		N = 307		N = 208		N = 221		N = 971	
**Total effects**										
ATT - > INT	−0.013	0.160	0.205∗∗	2.045	0.536∗∗∗	8.443	0.136∗∗	1.737	0.245∗∗∗	5.033
INT - > ABEH	0.059∗∗	1.762	0.072∗∗	2.274	0.102∗∗∗	3.691	0.096∗∗∗	2.970	0.092∗∗∗	5.579
PBC - > ABEH	0.244∗∗∗	9.987	0.241∗∗∗	9.319	0.186∗∗∗	6.799	0.150∗∗∗	4.902	0.224∗∗∗	17.066
PBC - > INT	0.375∗∗∗	5.179	0.165∗∗	1.984	0.077	1.133	0.080	1.253	0.188∗∗∗	5.057
PL - > INT	−0.015	0.336	0.008	0.222	−0.183∗∗∗	3.447	−0.098∗∗	1.786	−0.104∗∗∗	3.690
SN - > INT	0.414∗∗∗	5.688	0.502∗∗∗	4.542	0.070	0.960	0.553∗∗∗	6.775	0.357∗∗∗	6.474
R2 (INT)	0.453		0.595		0.448		0.476		0.492	
Q2 (INT)	0.426		0.554		0.411		0.454		0.481	
R2 (ABEH)	0.275		0.336		0.246		0.193		0.289	
Q2 (ABEH)	0.120		0.121		0.141		0.112		0.148	
**Indirect effects**										
ATT - > INT - > ABEH	−0.001	0.139	0.015	1.140	0.055∗∗∗	3.412	0.013∗	1.499	0.022∗∗∗	3.332
PBC - > INT - > ABEH	0.022∗∗	1.709	0.012∗	1.512	0.008	1.061	0.008	1.096	0.017∗∗∗	3.609
PL - > INT - > ABEH	−0.001	0.303	0.001	0.202	−0.019∗∗	2.254	−0.009∗	1.520	−0.010∗∗∗	2.895
SN - > INT - > ABEH	0.025∗∗	1.694	0.036∗∗∗	2.439	0.007	0.917	0.053∗∗∗	2.623	0.033∗∗∗	4.752

Note: O. sample denotes original sample, SD denotes standard deviation. ∗, ∗∗, and ∗∗∗ denotes significance at the 10 %, 5 %, and 1 % levels.

### 4.6 Impact of TPB constructs on intentions and actual adoption of PPT

Table 4 provides an overview of the effects of the constructs, on adoption intentions, intentions to expand the land area under PPT, and the actual adoption behavior. It presents path coefficients, t-values, and p-values, which serve as valuable indicators of the significance and strength of these impacts across the countries studied. Results presented in Table 4 (the reader is also referred to Figures B.1 to B.12), reveal that subjective norms, attitudes, and perceived behavioral control exhibited positive effects on adoption intentions, intention to expand the land area under PPT, and the actual adoption of the technology across the four countries. Perceived limitations had a negative effect on adoption intentions, intention to expand the land area under PPT, and the actual adoption of the technology across the four countries. Therefore, our hypotheses, which posit that farmers' subjective norms, attitudes, and perceived behavioral control positively influence their adoption intentions among non-users, as well as their expansion of land area allocated to the technology among current users, were confirmed. Additionally, it was confirmed that farmers' perceptions of PPT limitations negatively impact their adoption intentions among non-users and their expansion of land area allocated to PPT among current users. Moreover, our hypotheses regarding farmers’ behavioral intentions and perceived behavioral control positively impacting their actual PPT adoption behavior were also supported.

The literature argues that individuals are driven by either attitudes or subjective norms depending on the behavior being analyzed [35]. When it comes to adopting PPT, this study reveals that farmers in Rwanda and Tanzania are attitude driven, while farmers in Kenya and Uganda are more influenced by external social pressures than by their own internal perceptions (attitude) and their perceived capabilities to implement the technology (perceived behavioral control). When it comes to expanding the area of land allocated to PPT, farmers in Kenya are more motivated by the perception of their own abilities to implement the technology. In contrast, farmers in Uganda and Rwanda are more motivated by external social pressures, while farmers’ intentions in Tanzania are negatively impacted by their perceptions of the limitations of PPT.

#### 4.6.1 Attitudes

The statistically significant impact of attitudes on adoption intentions is well-established in the agricultural technology adoption literature. Numerous studies have identified attitudes as primary determinants of farmers' intentions. For instance, attitudes were the most influential factors in the implementation of integrated pest management in Serbia [40], formula fertilizer and soil testing technologies in China [34], agricultural production diversification practices in Brazil [59], food safety practices in Iran [36], and conservation agriculture in Bangladesh [43] and Mozambique [26]. In line with these findings, our study demonstrates that attitudes significantly influence farmers' decisions to adopt PPT, particularly in Rwanda and Tanzania, where they are the leading predictors of adoption intentions. Furthermore, attitudes significantly impact intentions to expand the land area under PPT in Kenya, Tanzania, and Uganda. Attitudes also indirectly influence the actual adoption of PPT in Tanzania and Uganda through their effect on intentions.

This significant impact suggests that farmers' positive assessment of the benefits and effectiveness of PPT plays a pivotal role in their intention to adopt or increase the land area allocated to the technology. Therefore, policy interventions that highlight the effectiveness and benefits of PPT and foster favorable perception of PPT among farmers could effectively enhance their adoption intentions and willingness to implement the technology. For example, one possible intervention could involve showcasing successful cases where other farmers have adopted PPT and benefitted from it. This strategy has been recommended in previous studies within the agricultural domain to increase farmers' implementation of innovative technologies [59]. Additionally, conducting economic impact assessments can reinforce positive attitudes by quantifying the benefits of PPT, thus encouraging wider adoption. A combination of these two approaches, i.e., disseminating success stories and evidence of economic benefits can enhance farmers' awareness and perceptions of PPT, ultimately leading to increased adoption rates and more extensive use of the technology.

#### 4.6.2 Subjective norms

It has been illustrated in adoption literature that individuals are not independent of social and cultural influences [35]. Therefore, they do not make decisions in isolation, but rather take into account how their actions align with those of others. Farmers are driven to adhere to social influences and are more likely to embrace new innovations when encouraged and supported by their fellow farmers [25,66]. Similarly, this study reveals that subjective norms significantly influence farmers' intentions to adopt PPT and expand the land area dedicated to this technology, particularly in Kenya, Uganda, and Rwanda. Moreover, this social influence extends beyond mere intentions, impacting the practical implementation of PPT. Farmers who perceive a high degree of social pressure are not only more likely to intend to adopt PPT but are also more likely to follow through with these intentions, resulting in actual adoption.

In Kenya and Uganda, subjective norms are the leading factor driving adoption intentions, while in Rwanda, they are the second most important determinant. For expanding the land area under PPT, subjective norms are the primary influence in Rwanda and Uganda, and the second most significant factor in Kenya. These findings imply that farmers experiencing greater social pressure or approval to adopt PPT are more inclined to follow through i.e., when farmers perceive that important figures in their social network endorse and approve the adoption of PPT, they are likely to experience increased motivation to conform to these expectations to gain social acceptance and avoid disapproval.

Empirical studies provide robust support for this finding. For instance, research by Bagheri et al. [42] demonstrated that subjective norms were a strong predictor of the intentions of Iranian farmers to implement integrated crop management (ICM) practices. Similarly, Castillo et al. [41] reported that social approval influenced significantly Chilean farmers' adoption of pressurized irrigation technologies. Drescher et al. [52] also noted that Canadian (Ontario) farmers' decisions to adopt environmental best management practices were heavily swayed by subjective norms. Kamau et al. [66] also reported that social norms greatly influenced the uptake of improved breeds of indigenous chicken among Kenyan farmers.

Therefore, interventions aimed at involving extension officers, fellow farmers, family members, and farmer groups or cooperatives in the decision-making process regarding PPT adoption would effectively heighten social pressure on farmers, thereby improving their intentions to adopt the technology. Moreover, the significant impact of subjective norms on intentions obtained in this study could be attributed to the continuous efforts undertaken to promote the adoption of PPT, effectively increasing social pressure on farmers to adopt the technology. This also reflects the efficacy of learning from peers and observational learning techniques facilitated through the exchange of knowledge among farmers. These methods, along with other participatory approaches that can be tailored to suit specific local farming situations, have proven to be highly beneficial in the uptake of innovations [25,66].

#### 4.6.3 Perceived behavioral control

Examining the impact of perceived behavioral control on PPT adoption intentions is important because PPT is regarded as a knowledge-intensive technology, with its adoption heavily dependent on access to information through effective channels [14,67]. In addition to information, access to essential inputs, such as companion plants, is crucial. Therefore, having adequate knowledge about the technology and access to the necessary inputs are critical factors for its successful adoption. Moreover, although agricultural advisors and extension officers are available in the study areas, not all farmers engage with them. Consequently, these farmers may lack the competence or confidence to implement the technology effectively.

The results indicate that perceived behavioral control significantly influences intentions to implement PPT. Additionally, this construct plays a pivotal role in the actual adoption of PPT across the four countries studied. Perceived behavioral control notably impacts adoption intentions in Kenya, Rwanda, and Uganda, as well as intentions to expand the PPT land area in Kenya, Tanzania, and Uganda. These findings indicate that farmers' perceptions regarding the ease of adopting PPT, their self-confidence in executing the adoption, the degree of control they possess over the adoption process, and the availability of necessary inputs are pivotal factors in determining their intentions to adopt PPT and increase the land area allocated to this technology.

While perceived behavioral control may have a smaller effect on farmers' adoption intentions compared to their attitudes and subjective norms^3^, this construct exhibits a significant direct influence on the actual PPT adoption, surpassing the effect of behavioral intention across all four countries studied. This emphasizes the critical importance of enhancing farmers' abilities to implement PPT by providing the essential resources, including information and inputs, to enhance adoption. The significant impact of this component aligns with the results of previous research that have highlighted the relevance of perceived behavioral control in agricultural technology adoption contexts. For instance, Daxini et al. [46] reported a positive significant impact of perceived behavioral control on intentions to adopt a nutrient management plan. Similarly, Omulo et al. [25] revealed that perceived behavioral control significantly influenced farmers' intentions to adopt mechanized conservation agriculture. Nguyen and Drakou [44] also identified a strong positive effect of perceived behavioral control on the adoption of sustainable agricultural practices.

These results suggest that interventions aimed at increasing farmers' access to resources, such as training, technical support, and input availability, could significantly enhance PPT adoption rates. By improving farmers' confidence in their ability to adopt PPT and providing them with greater control over the adoption process, these interventions can address key barriers to adoption. For example, targeted training programs that equip farmers with practical skills and knowledge about PPT, along with ensuring the availability of necessary inputs, can substantially boost their perceived behavioral control and consequently their adoption intentions and actions. Moreover, the direct effect of perceived behavioral control on actual adoption underscores the necessity of designing policies and programs that not only inform farmers about the benefits of PPT but also actively support them in overcoming practical challenges related to adoption. This dual approach of raising awareness and providing tangible support can create a more conducive environment for the widespread adoption of PPT.

#### 4.6.4 Perceived limitations

Perceived limitations significantly and negatively affect farmers’ intentions to adopt PPT in Rwanda and Uganda. Regarding intentions to expand the land area dedicated to PPT, perceived limitations are significant only in Tanzania, where they hold the highest importance among the TPB constructs. Furthermore, perceived limitations indirectly influence the actual adoption of PPT in Tanzania and Uganda through their impact on intentions. This highlights the critical role of barriers such as labor requirements and initial costs in these countries. Notably, perceived limitations generally exert the least influence on behavioral intentions compared to other constructs except among users in Tanzania. This suggests that while perceived limitations are significant, their influence is often secondary to other motivational factors. Farmers may still be inclined to adopt PPT or increase the land area under PPT if they hold positive attitudes towards its benefits, possess a high level of self-confidence in their capability to implement PPT, trust in their peers' opinions, and have minimal concerns about the limitations of the technology. This insight underscores the necessity of addressing farmers' perceptions and attitudes when promoting the adoption of PPT, thereby enhancing its perceived benefits and feasibility. For instance, providing detailed information on the long-term economic benefits of PPT can help offset concerns about initial costs and labor requirements. Demonstrating flexibility in PPT implementation through success stories and adaptable practices can further alleviate concerns about the limited flexibility of the technology.

#### 4.6.5 Intentions

Our findings provide compelling evidence of the pivotal role of intentions in directly influencing farmers’ actual adoption of PPT across all four countries studied. This aligns well with the foundational tenets of the Theory of Planned Behavior, which suggests that intentions serve as immediate precursors to actual behavior. The theory suggests that individuals who harbor strong intentions to engage in a specific behavior are more inclined to carry it out and manifest that behavior in practice [28]. This result underscores the practical importance of understanding and addressing farmers' intentions when creating strategies to encourage the adoption of PPT. Farmers who exhibit a firm commitment and resolve to adopt PPT are not only predisposed to initiate the adoption process but are also more inclined to overcome potential barriers and actively implement the technology on their farms.

Recognizing the significance of intentions in driving adoption behavior further highlights the need for targeted interventions that aim to enhance farmers' intentions through tailored promotion strategies, capacity-building initiatives, and support systems. Promotion efforts should be tailored to align with the specific needs, preferences, and perceptions of the farmers, thereby maximizing their impact on intention formation. Initiatives such as training programs can empower farmers with the requisite knowledge and competencies to translate their intentions into action. Additionally, establishing supportive networks and community structures can provide farmers with the social reinforcement and resources needed to sustain their intentions and navigate the adoption process effectively.

### 4.7 ANN analysis results

We extend this study's analysis by utilizing ANN to validate the PLS-SEM estimates. Drawing from previous research i.e., Barua and Barua [53], Zhou et al. [50], Chahal et al. [55], Wang et al. [49], and Leong et al. [54], a ten-fold cross-validation method was employed to ensure accurate and reliable findings. The dataset was divided into a 90 % training set and the remaining 10 % for testing, thereby mitigating the risk of over-fitting [53]. To evaluate the significance of predictor variables, we utilized a Multilayer Perceptron (MLP) for both training and testing in all models. To effectively represent the continuous function, the current study incorporated a single hidden layer. The activation function chosen for the hidden and output layers was the sigmoid function. The number of hidden neurons was determined automatically. The models' precision was evaluated by calculating the RMSE (root mean square of error) values derived from the 10 networks for each model. The ANN models' predictive accuracy was estimated by averaging the obtained RMSE values. Table 5 presents the mean and standard deviation of the 10 ANNs estimated for training and testing sets. These values show minimal variation in the mean RMSE values for both the training and testing phases. These denote the consistency of ANN models in generating accurate relationships expressed numerically between predictors and outputs [53]. The low average RMSE values further highlight the robustness of the models [55].

**Table 5:** Root mean square of error values.

		Non-users		Users		Pooled sample	
		Mean	SD	Mean	SD	Mean	SD
Training							
Kenya	SSE	1.464	0.480	1.512	0.652	2.243	0.342
	RMSE	0.124	0.020	0.111	0.018	0.104	0.008
Rwanda	SSE	1.145	0.289	1.067	0.223	1.716	0.132
	RMSE	0.108	0.013	0.077	0.008	0.078	0.002
Tanzania	SSE	1.355	0.179	0.824	0.258	1.841	0.400
	RMSE	0.103	0.007	0.112	0.017	0.099	0.010
Uganda	SSE	2.340	0.359	0.871	0.292	3.425	0.700
	RMSE	0.134	0.009	0.110	0.016	0.130	0.012
All countries combined	SSE	5.435	0.509	2.619	0.096	6.514	0.237
	RMSE	0.110	0.005	0.078	0.001	0.086	0.002
**Testing**							
Kenya	SSE	0.185	0.194	0.117	0.052	0.295	0.143
	RMSE	0.115	0.057	0.092	0.022	0.103	0.025
Rwanda	SSE	0.098	0.048	0.107	0.078	0.150	0.074
	RMSE	0.086	0.016	0.070	0.022	0.071	0.012
Tanzania	SSE	0.078	0.061	0.075	0.084	0.164	0.066
	RMSE	0.085	0.023	0.109	0.052	0.087	0.014
Uganda	SSE	0.217	0.080	0.041	0.026	0.275	0.066
	RMSE	0.122	0.018	0.069	0.018	0.114	0.019
All countries combined	SSE	0.563	0.237	0.249	0.083	0.758	0.166
	RMSE	0.105	0.017	0.071	0.011	0.087	0.009

**Note:** SD denotes standard deviation, SSE denotes the Sum square of errors, RMSE denotes the Root mean square of errors.

#### 4.7.1 Sensitivity analysis

A sensitivity analysis was conducted to determine the relative significance of the predictors. This analysis involved calculating the normalized relative importance of each variable in each model. The normalized importance was calculated by dividing the relative importance of each predictor variable by the highest relative importance among all the variables in each ANN model. This allows for a standardized assessment of importance across different predictors [49,54]. The results presented in Table 6 align with the outcomes of the PLS-SEM analysis, providing further validity to the results. For instance, in Kenya, perceived behavioral control and subjective norms are the most critical factors influencing intentions to adopt and expand the PPT land area. Conversely, in Rwanda, subjective norms and attitudes play key roles in determining intentions to adopt and expand the land area under PPT. Meanwhile, attitudes stand out as the primary determinant of farmers' intentions to adopt and expand the land area under PPT in Tanzania. In Uganda, subjective norms take precedence as the primary determinants of adoption intentions and intentions to expand the land area under PPT.

**Table 6:** Sensitivity analysis.

		Non-users			Users			Pooled sample		
	Construct	Av.import	Norm. Imp (%)	Rank	Av.import	Norm. Imp (%)	Rank	Av.import	Norm. Imp (%)	Rank
Kenya	ATT	0.146	35.74	3	0.239	70.12	3	0.096	21.47	3
	PL	0.125	32.12	4	0.101	28.54	4	0.029	6.14	4
	PBC	0.312	76.92	2	0.342	100.00	1	0.384	78.74	2
	SN	0.417	100.00	1	0.318	90.39	2	0.491	100.00	1
Rwanda	ATT	0.286	91.99	2	0.135	24.48	3	0.290	62.33	2
	PL	0.198	64.65	3	0.058	9.10	4	0.033	7.42	4
	PBC	0.190	61.99	4	0.193	32.95	2	0.195	43.09	3
	SN	0.326	100.00	1	0.614	100.00	1	0.483	100.00	1
Tanzania	ATT	0.484	100.00	1	0.337	100.00	1	0.561	100.00	1
	PL	0.152	37.18	4	0.220	66.98	3	0.214	45.55	2
	PBC	0.167	42.26	3	0.285	82.68	2	0.100	21.42	4
	SN	0.198	48.26	2	0.157	48.38	4	0.126	30.65	3
Uganda	ATT	0.211	53.41	3	0.323	80.66	2	0.290	68.04	2
	PL	0.156	37.53	4	0.078	19.63	4	0.074	17.30	4
	PBC	0.222	55.11	2	0.194	49.05	3	0.198	50.02	3
	SN	0.412	100.00	1	0.406	100.00	1	0.439	100.00	1
All countries combined	ATT	0.296	79.65	2	0.234	55.21	3	0.310	77.50	2
	PL	0.119	33.79	4	0.035	8.29	4	0.089	22.28	4
	PBC	0.207	56.98	3	0.294	68.87	2	0.195	49.77	3
	SN	0.378	100.00	1	0.436	100.00	1	0.407	100.00	1

**Note:** SN denotes Subjective Norms, PBC denotes Perceived Behavioral Control, ATT denotes Attitudes and PL denotes Perceived Limitations, Av. Imp. denotes Average Importance, Norm. Imp. denotes Normalized Importance.

## 5 Conclusion

Since its inception in the 1990s, push-pull technology has demonstrated effectiveness in the control of striga weed, stemborers, and fall armyworms. Moreover, the technology is recognized as an economical method of conservation agriculture, capable of doubling or even tripling cereal yields, improving soil fertility, and providing fodder for livestock. Despite these benefits, adoption rates remain low, even with significant promotional efforts. This study extends the PPT adoption literature by shifting focus from the commonly examined socioeconomic factors to social-psychological factors impacting the adoption of PPT, guided by the Theory of Planned Behavior (TPB). Moreover, this study extends the application of the TPB in agricultural settings by incorporating the construct of "Perceived Limitations" and examining its role alongside traditional TPB elements, attitudes, subjective norms, and perceived behavioral control in shaping farmers' intentions to adopt or expand the use of PPT. The PLS-SEM and ANN are used to analyze three models: one for non-users assessing their intentions to adopt push-pull technology, another for current users examining their intentions to expand the land area under push-pull technology, and a third model using a pooled sample to evaluate the impact of TPB constructs on actual adoption.

This study unveils a high level of intention to adopt or expand the land area under PPT among farmers, despite its relatively current low adoption rate. This intention is primarily driven by the four constructs considered in this study, i.e., positive attitudes of farmers towards PPT, subjective norms, their perceptions regarding their capability to successfully adopt this technology, and their perceptions of the limitations of PPT. While farmers perceive PPT as a valuable and beneficial technology for enhancing improved productivity and overall profitability on their farms, their adoption decisions are also influenced by the opinions, recommendations, and experiences shared by their peers, agricultural experts, family members, and other influential individuals within their community. Additionally, farmers who believe in their skills, knowledge, and resources to successfully implement and utilize PPT are more likely to adopt or expand their land use area under the technology. On the other hand, farmers who perceive barriers and challenges in adopting PPT may have a lower intention to adopt or increase the land area under the technology. Moreover, these components also significantly impact the actual adoption.

These findings underscore the necessity of considering farmers' social psychological attributes when designing and implementing PPT dissemination programs. This ensures that these programs are more effective and better aligned with the needs and perceptions of the farming community. A holistic approach that addresses these multiple dimensions, i.e., enhancing farmers’ overall evaluation of the benefits and effectiveness of the technology, social influences, and their confidence in their ability to implement the technology through training and support services, while also addressing the limitations of the technology could be essential in enhancing widespread adoption.

The study results further reveal significant variations in the effect of TPB constructs on adoption intentions and intentions to expand land allocated to PPT across the studied countries. In Kenya and Uganda, while subjective norms and farmers' confidence in their ability to adopt PPT are the most critical determinants of adoption intentions, attitudes also significantly influence the intentions to expand the land area allocated to PPT. In Rwanda, while all four constructs significantly influence adoption intentions, only subjective norms significantly influence the intentions to expand the land area allocated to PPT. In Tanzania, while farmers' perceptions of the limitations of PPT, their attitudes, and perceived behavioral control significantly influence their intentions to expand the land area under PPT, only attitudes significantly influence adoption intentions. Therefore, tailoring intervention strategies to specific determinants relevant in each country could be necessary to promote more effective and widespread adoption.

Our findings further reveal several key insights that broaden the TPB's relevance in agricultural decision-making contexts. Firstly, the significant negative influence of "perceived limitations" on farmers' intentions to adopt or expand PPT demonstrates the importance of considering contextual and technology-specific constraints within the TPB framework. This extension provides a more nuanced understanding of adoption behavior by recognizing that practical limitations such as labor demands, crop rotation inflexibility, and financial costs can moderate the impact of farmers' intentions, even when attitudes, subjective norms, and perceived behavioral control are favorable. This insight highlights that in agricultural settings, especially in low-resource regions, the perceived feasibility of adopting technology can be as influential as the desire to adopt it, suggesting that TPB applications in agriculture can benefit from constructs that capture practical and context-dependent constraints.

Secondly, this study's cross-country analysis shows that the strength and direction of TPB constructs vary by country, underscoring the importance of adapting TPB-based models to the specific social, economic, and agricultural conditions within different regions. These findings suggest that the TPB framework, when adapted to include both behavioral and contextual factors like "perceived limitations," can offer valuable insights into country-specific determinants of technology adoption. This contextual sensitivity adds a unique layer to TPB's applicability in agriculture by showing that policies and interventions aiming to promote adoption must be responsive not only to general attitudes or norms but also to the localized perceptions and constraints that shape behavior in diverse agricultural settings.

## 6 Limitations of the study

Our study aimed to identify the effects of attitude, subjective norm, perceived behavioral control, and perceived limitations on farmers' intentions to implement PPT or expand the land area under this technology. To achieve this, we focused on the unidirectional causality of the TPB constructs on intentions. However, previous research, such as Daxini et al. [35], has demonstrated causal interrelationships among the TPB constructs. For instance, subjective norm was found to be an important predictor of both attitude and perceived behavioral control, in addition to its impact on intention. Future studies could explore these interrelationships in the context of PPT. While our overall sample size was relatively large, the sample sizes at the country level were small, limiting the generalizability of our findings to broader populations. This study only examined social psychological factors, as socioeconomic variables were found to be insignificant. Future research could include policy and institutional variables to enhance the explanatory power of the models and provide more robust results.

Additionally, our study utilized cross-sectional data. Longitudinal studies would be valuable for capturing how farmers' attitudes, norms, perceived control, and perceived limitations evolve as they gain experience with PPT. Tracking these changes over time could provide insights into whether perceived limitations decrease as familiarity with the technology grows or if other barriers emerge as adoption expands. This dynamic approach would help refine the TPB framework in agricultural contexts by highlighting how intention-related factors change with farmers’ experiences and the progression of technology adoption.

## CRediT authorship contribution statement

**Denis Waiswa:** Writing – review & editing, Writing – original draft, Visualization, Validation, Software, Methodology, Investigation, Formal analysis, Data curation, Conceptualization. **Beatrice Wambui Muriithi:** Writing – review & editing, Validation, Supervision, Resources, Project administration, Investigation, Funding acquisition, Conceptualization. **Alice W. Murage:** Writing – review & editing, Validation, Supervision, Resources, Project administration, Investigation, Funding acquisition, Conceptualization. **Dave Mwangi Ireri:** Writing – review & editing, Validation, Investigation, Data curation, Conceptualization. **Fredah Maina:** Writing – review & editing, Validation, Supervision, Resources, Project administration, Investigation, Funding acquisition, Conceptualization. **Frank Chidawanyika:** Writing – review & editing, Validation, Resources, Project administration, Investigation, Funding acquisition, Conceptualization. **Fahri Yavuz:** Writing – review & editing, Validation, Supervision, Conceptualization.

## Data availability

The datasets generated during and/or analyzed during the current study are available from the corresponding author on reasonable request.

## Ethics declarations

This study was reviewed and approved by Jomo Kenyatta University of Agriculture and Technology Institutional Ethics Review Committee with the approval number: JKU/IERC/02316/0104, dated May 13, 2021. Verbal informed consent was obtained prior to the interview.

## Funding

This research was funded by the 10.13039/501100000780European Union (EU) H2020-SFS-2018-2020/H2020-SFS-2019-2, Grant Number 861998.

## Declaration of competing interest

The authors declare that they have no known competing financial interests or personal relationships that could have appeared to influence the work reported in this paper.

## Footnotes

1 Among the eight intention statements, INT1, INT2, INT3, INT7, and INT8 were addressed by the entire sample (both users and non-users). In contrast, INT4, INT5, and INT6 were answered only by non-users. Consequently, three models were estimated: one for users, one for non-users, and one for the pooled sample.

## Footnotes

2 The maps are based on 2023 land use or land cover data downloaded from https://livingatlas.arcgis.com/landcover/.

## Footnotes

3 Perceived behavioral control stands out in Kenya as the primary predictor of intentions to expand the PPT land area.
