# Supplementary material for: The role of social-psychological factors in the adoption of push-pull technology by small-scale farmers in East Africa: Application of the theory of planned behavior
Source: Heliyon. 2024 Dec 26;11(1):e41449. doi: 10.1016/j.heliyon.2024.e41449 (PMC11762192; doi:10.1016/j.heliyon.2024.e41449)
Supplement: Multimedia component 1 [file mmc1.docx]

**Supplementary material for the paper:**

**The Role of Social-Psychological Factors in the Adoption of Push-Pull Technology by Small-scale Farmers in East Africa: Application of the Theory of Planned Behavior**

**Appendix A: Tables**

**Table A.1.** Reliability of the measurement models

|  | | Non-users | | | | Users | | | | Pooled sample | | | |
| --- | --- | --- | --- | --- | --- | --- | --- | --- | --- | --- | --- | --- | --- |
|  |  | α | rho_a | rho_c | AVE | α | rho_a | rho_c | AVE | α | rho_a | rho_c | AVE |
| Kenya | ATT | 0.93 | 0.94 | 0.93 | 0.52 | 0.77 | 0.78 | 0.84 | 0.52 | 0.92 | 0.93 | 0.93 | 0.51 |
|  | INT | 0.82 | 0.91 | 0.88 | 0.64 | 0.71 | 0.71 | 0.82 | 0.53 | 0.77 | 0.77 | 0.90 | 0.81 |
|  | PBC | 0.88 | 0.93 | 0.91 | 0.59 | 0.81 | 0.81 | 0.86 | 0.52 | 0.89 | 0.90 | 0.91 | 0.60 |
|  | PL | 0.78 | 0.80 | 0.84 | 0.64 |  |  |  |  | 0.77 | 0.48 | 0.81 | 0.59 |
|  | SN | 0.87 | 0.88 | 0.90 | 0.55 | 0.81 | 0.83 | 0.86 | 0.51 | 0.87 | 0.88 | 0.90 | 0.56 |
| Rwanda | ATT | 0.91 | 0.91 | 0.92 | 0.52 | 0.84 | 0.84 | 0.88 | 0.51 | 0.92 | 0.92 | 0.93 | 0.51 |
|  | INT | 0.84 | 0.86 | 0.88 | 0.51 | 0.87 | 0.87 | 0.90 | 0.65 | 0.84 | 0.88 | 0.88 | 0.60 |
|  | PBC | 0.86 | 0.88 | 0.89 | 0.51 | 0.82 | 0.84 | 0.87 | 0.53 | 0.89 | 0.90 | 0.91 | 0.57 |
|  | PL | 0.76 | 0.83 | 0.86 | 0.68 | 0.87 | 0.92 | 0.91 | 0.71 | 0.88 | 0.98 | 0.91 | 0.68 |
|  | SN | 0.85 | 0.86 | 0.88 | 0.52 | 0.86 | 0.88 | 0.89 | 0.55 | 0.88 | 0.89 | 0.91 | 0.59 |
| Tanzania | ATT | 0.94 | 0.95 | 0.95 | 0.62 | 0.90 | 0.92 | 0.92 | 0.50 | 0.94 | 0.95 | 0.95 | 0.60 |
|  | INT | 0.79 | 0.84 | 0.84 | 0.52 | 0.77 | 0.80 | 0.85 | 0.60 | 0.80 | 0.81 | 0.86 | 0.55 |
|  | PBC | 0.85 | 0.92 | 0.86 | 0.52 | 0.83 | 0.86 | 0.88 | 0.55 | 0.86 | 0.88 | 0.89 | 0.55 |
|  | PL | 0.81 | 0.96 | 0.86 | 0.63 | 0.86 | 0.87 | 0.90 | 0.65 | 0.85 | 0.86 | 0.89 | 0.63 |
|  | SN | 0.73 | 0.76 | 0.80 | 0.51 | 0.79 | 0.82 | 0.86 | 0.54 | 0.77 | 0.78 | 0.84 | 0.51 |
| Uganda | ATT | 0.95 | 0.96 | 0.96 | 0.64 | 0.77 | 0.77 | 0.82 | 0.31 | 0.94 | 0.94 | 0.94 | 0.57 |
|  | INT | 0.80 | 0.83 | 0.86 | 0.56 | 0.67 | 0.78 | 0.79 | 0.49 | 0.72 | 0.76 | 0.88 | 0.78 |
|  | PBC | 0.86 | 0.87 | 0.90 | 0.56 | 0.78 | 0.86 | 0.85 | 0.54 | 0.86 | 0.87 | 0.90 | 0.56 |
|  | PL | 0.79 | 0.81 | 0.86 | 0.62 | 0.73 | 0.81 | 0.82 | 0.61 | 0.80 | 0.82 | 0.88 | 0.71 |
|  | SN | 0.86 | 0.90 | 0.90 | 0.60 | 0.75 | 0.76 | 0.84 | 0.51 | 0.90 | 0.91 | 0.92 | 0.63 |
| All countries combined | ATT | 0.95 | 0.95 | 0.95 | 0.59 | 0.81 | 0.83 | 0.86 | 0.51 | 0.94 | 0.94 | 0.95 | 0.54 |
|  | INT | 0.74 | 0.77 | 0.83 | 0.50 | 0.77 | 0.78 | 0.86 | 0.60 | 0.77 | 0.81 | 0.85 | 0.58 |
|  | PBC | 0.86 | 0.88 | 0.89 | 0.54 | 0.78 | 0.79 | 0.85 | 0.53 | 0.88 | 0.89 | 0.90 | 0.54 |
|  | PL | 0.71 | 0.71 | 0.82 | 0.53 | 0.85 | 0.85 | 0.89 | 0.62 | 0.83 | 0.89 | 0.88 | 0.59 |
|  | SN | 0.86 | 0.87 | 0.90 | 0.56 | 0.82 | 0.82 | 0.87 | 0.52 | 0.88 | 0.89 | 0.91 | 0.59 |

**Note:** α denotes Cronbach's alpha, AVE denotes the Average variance extracted, CR denotes Composite Reliability

**Table A.2.** Discriminant validity - Heterotrait-monotrait ratio (HTMT)

|  | Kenya | Rwanda | Tanzania | Uganda | All countries combined |
| --- | --- | --- | --- | --- | --- |
| **Non-users** | | | | | |
| INT <-> ATT | 0.22 | 0.70 | 0.57 | 0.53 | 0.54 |
| PBC <-> ATT | 0.38 | 0.49 | 0.49 | 0.65 | 0.54 |
| PBC <-> INT | 0.41 | 0.51 | 0.23 | 0.65 | 0.47 |
| PL <-> ATT | 0.18 | 0.25 | 0.14 | 0.77 | 0.26 |
| PL <-> INT | 0.11 | 0.44 | 0.19 | 0.35 | 0.10 |
| PL <-> PBC | 0.18 | 0.27 | 0.16 | 0.57 | 0.29 |
| SN <-> ATT | 0.51 | 0.73 | 0.55 | 0.85 | 0.71 |
| SN <-> INT | 0.48 | 0.72 | 0.37 | 0.73 | 0.66 |
| SN <-> PBC | 0.31 | 0.59 | 0.49 | 0.83 | 0.60 |
| SN <-> PL | 0.11 | 0.26 | 0.29 | 0.68 | 0.17 |
| **Users** | | | | | |
| INT <-> ATT | 0.76 | 0.43 | 0.51 | 0.67 | 0.52 |
| PBC <-> ATT | 0.63 | 0.51 | 0.17 | 0.44 | 0.46 |
| PBC <-> INT | 0.90 | 0.53 | 0.45 | 0.52 | 0.64 |
| PL <-> ATT | 0.15 | 0.13 | 0.24 | 0.26 | 0.09 |
| PL <-> INT | 0.27 | 0.06 | 0.67 | 0.24 | 0.14 |
| PL <-> PBC | 0.12 | 0.17 | 0.35 | 0.28 | 0.16 |
| SN <-> ATT | 0.53 | 0.49 | 0.50 | 0.70 | 0.54 |
| SN <-> INT | 0.85 | 0.77 | 0.55 | 0.75 | 0.76 |
| SN <-> PBC | 0.72 | 0.60 | 0.29 | 0.53 | 0.59 |
| SN <-> PL | 0.36 | 0.12 | 0.48 | 0.32 | 0.17 |
| **Pooled sample** | | | | | |
| ATT <-> ABEH | 0.53 | 0.54 | 0.32 | 0.36 | 0.45 |
| INT <-> ABEH | 0.43 | 0.42 | 0.37 | 0.41 | 0.43 |
| INT <-> ATT | 0.52 | 0.68 | 0.72 | 0.66 | 0.68 |
| PBC <-> ABEH | 0.55 | 0.60 | 0.48 | 0.44 | 0.55 |
| PBC <-> ATT | 0.62 | 0.68 | 0.46 | 0.67 | 0.64 |
| PBC <-> INT | 0.69 | 0.63 | 0.40 | 0.67 | 0.63 |
| PL <-> ABEH | 0.09 | 0.20 | 0.18 | 0.13 | 0.08 |
| PL <-> ATT | 0.13 | 0.27 | 0.17 | 0.56 | 0.08 |
| PL <-> INT | 0.05 | 0.20 | 0.30 | 0.22 | 0.11 |
| PL <-> PBC | 0.16 | 0.21 | 0.12 | 0.31 | 0.08 |
| SN <-> ABEH | 0.41 | 0.46 | 0.41 | 0.44 | 0.48 |
| SN <-> ATT | 0.66 | 0.72 | 0.70 | 0.83 | 0.76 |
| SN <-> INT | 0.71 | 0.83 | 0.57 | 0.82 | 0.74 |
| SN <-> PBC | 0.56 | 0.69 | 0.56 | 0.81 | 0.70 |
| SN <-> PL | 0.07 | 0.24 | 0.34 | 0.41 | 0.06 |

**Table A.3.** Discriminant validity - Fornell-Larcker criterion for the models

|  | | Non-users | | | | | Users | | | | | Pooled sample | | | | | |
| --- | --- | --- | --- | --- | --- | --- | --- | --- | --- | --- | --- | --- | --- | --- | --- | --- | --- |
|  |  | ATT | INT | PBC | PL | SN | ATT | INT | PBC | PL | SN | ABEH | ATT | INT | PBC | PL | SN |
| Kenya | ABEH |  |  |  |  |  |  |  |  |  |  | 1.000 |  |  |  |  |  |
|  | ATT | 0.722 |  |  |  |  | 0.718 |  |  |  |  | 0.515 | 0.711 |  |  |  |  |
|  | INT | 0.224 | 0.803 |  |  |  | 0.577 | 0.731 |  |  |  | 0.375 | 0.449 | 0.900 |  |  |  |
|  | PBC | 0.387 | 0.378 | 0.765 |  |  | 0.51 | 0.683 | 0.718 |  |  | 0.515 | 0.573 | 0.572 | 0.776 |  |  |
|  | PL | 0.03 | -0.057 | -0.033 | 0.801 |  | -0.129 | -0.232 | -0.099 | 1 |  | 0.083 | 0.122 | 0.059 | 0.153 | 0.771 |  |
|  | SN | 0.484 | 0.458 | 0.303 | 0.019 | 0.745 | 0.448 | 0.675 | 0.599 | -0.331 | 0.716 | 0.393 | 0.602 | 0.593 | 0.500 | 0.043 | 0.750 |
| Rwanda | ABEH |  |  |  |  |  |  |  |  |  |  | 1.000 |  |  |  |  |  |
|  | ATT | 0.722 |  |  |  |  | 0.714 |  |  |  |  | 0.516 | 0.712 |  |  |  |  |
|  | INT | 0.649 | 0.714 |  |  |  | 0.374 | 0.809 |  |  |  | 0.436 | 0.633 | 0.778 |  |  |  |
|  | PBC | 0.445 | 0.479 | 0.714 |  |  | 0.422 | 0.467 | 0.725 |  |  | 0.567 | 0.615 | 0.598 | 0.757 |  |  |
|  | PL | -0.211 | -0.379 | -0.157 | 0.824 |  | -0.084 | 0.039 | 0.035 | 0.844 |  | -0.191 | -0.249 | -0.187 | -0.177 | 0.823 |  |
|  | SN | 0.658 | 0.654 | 0.526 | -0.229 | 0.721 | 0.425 | 0.686 | 0.501 | -0.101 | 0.741 | 0.434 | 0.653 | 0.734 | 0.613 | -0.226 | 0.767 |
| Tanzania | ABEH |  |  |  |  |  |  |  |  |  |  | 1.000 |  |  |  |  |  |
|  | ATT | 0.79 |  |  |  |  | 0.708 |  |  |  |  | 0.316 | 0.774 |  |  |  |  |
|  | INT | 0.559 | 0.723 |  |  |  | 0.471 | 0.772 |  |  |  | 0.343 | 0.634 | 0.742 |  |  |  |
|  | PBC | 0.457 | 0.276 | 0.719 |  |  | 0.001 | 0.386 | 0.74 |  |  | 0.452 | 0.421 | 0.346 | 0.743 |  |  |
|  | PL | -0.012 | 0.095 | 0.093 | 0.793 |  | -0.157 | -0.565 | -0.301 | 0.804 |  | -0.163 | -0.114 | -0.260 | -0.042 | 0.792 |  |
|  | SN | 0.444 | 0.313 | 0.407 | -0.106 | 0.713 | 0.444 | 0.464 | 0.256 | -0.392 | 0.737 | 0.368 | 0.636 | 0.492 | 0.501 | -0.232 | 0.717 |
| Uganda | ABEH |  |  |  |  |  |  |  |  |  |  | 1.000 |  |  |  |  |  |
|  | ATT | 0.799 |  |  |  |  | 0.559 |  |  |  |  | 0.343 | 0.756 |  |  |  |  |
|  | INT | 0.489 | 0.748 |  |  |  | 0.585 | 0.701 |  |  |  | 0.358 | 0.559 | 0.883 |  |  |  |
|  | PBC | 0.61 | 0.558 | 0.747 |  |  | 0.297 | 0.403 | 0.736 |  |  | 0.405 | 0.606 | 0.531 | 0.747 |  |  |
|  | PL | 0.682 | 0.31 | 0.469 | 0.785 |  | 0.046 | -0.182 | -0.17 | 0.781 |  | 0.114 | 0.491 | 0.184 | 0.261 | 0.844 |  |
|  | SN | 0.778 | 0.635 | 0.724 | 0.571 | 0.778 | 0.542 | 0.646 | 0.418 | -0.185 | 0.717 | 0.409 | 0.766 | 0.677 | 0.712 | 0.355 | 0.791 |
| All countries combined | ABEH |  |  |  |  |  |  |  |  |  |  | 1.000 |  |  |  |  |  |
|  | ATT | 0.769 |  |  |  |  | 0.715 |  |  |  |  | 0.440 | 0.736 |  |  |  |  |
|  | INT | 0.472 | 0.704 |  |  |  | 0.42 | 0.773 |  |  |  | 0.409 | 0.598 | 0.762 |  |  |  |
|  | PBC | 0.509 | 0.398 | 0.738 |  |  | 0.374 | 0.498 | 0.73 |  |  | 0.515 | 0.584 | 0.548 | 0.735 |  |  |
|  | PL | 0.204 | 0.044 | 0.227 | 0.728 |  | -0.034 | -0.115 | -0.044 | 0.787 |  | -0.083 | 0.033 | -0.102 | 0.028 | 0.766 |  |
|  | SN | 0.648 | 0.537 | 0.537 | 0.13 | 0.746 | 0.441 | 0.62 | 0.472 | -0.139 | 0.721 | 0.450 | 0.693 | 0.645 | 0.622 | -0.034 | 0.766 |

**Table A.4.** Variance Inflation Factors for the variables in each model

|  | Non-users | | | | | Users | | | | | Pooled sample | | | | |
| --- | --- | --- | --- | --- | --- | --- | --- | --- | --- | --- | --- | --- | --- | --- | --- |
|  | Kenya | Rwanda | Tanzania | Uganda | All countries combined | Kenya | Rwanda | Tanzania | Uganda | All countries combined | Kenya | Rwanda | Tanzania | Uganda | All countries combined |
| ABEH |  |  |  |  |  |  |  |  |  |  | 1.00 | 1.00 | 1.00 | 1.00 | 1.00 |
| ATT1 | 2.18 | 4.66 | 4.14 | 3.33 | 3.91 |  |  | 2.60 | 2.49 |  | 2.05 | 2.75 | 3.82 | 2.55 | 3.11 |
| ATT2 | 2.61 | 3.98 | 3.99 |  | 4.12 |  |  | 2.52 | 2.72 |  | 2.20 | 2.57 | 3.37 |  | 3.18 |
| ATT3 | 3.10 |  | 2.14 | 3.44 | 2.20 |  |  | 2.56 | 1.33 |  | 2.29 |  | 2.22 | 2.58 | 2.13 |
| ATT4 | 2.84 |  | 2.85 | 3.21 | 2.50 |  |  |  |  |  | 2.17 | 1.90 | 2.69 | 2.25 | 2.14 |
| ATT5 | 3.39 | 2.18 | 4.86 | 3.76 | 2.79 |  | 1.53 | 3.75 | 1.42 | 1.28 | 3.01 | 1.99 | 4.02 | 2.91 | 2.39 |
| ATT6 | 2.59 | 3.32 |  | 4.95 | 3.93 |  | 2.53 | 2.33 | 2.79 | 2.21 | 2.22 | 3.13 |  | 3.69 | 3.38 |
| ATT7 | 2.73 | 3.44 | 3.26 | 3.43 | 3.26 |  | 2.29 |  | 2.74 | 2.02 | 2.65 | 3.01 | 2.22 | 3.17 | 3.01 |
| ATT8 | 2.62 | 2.07 |  | 4.73 | 3.47 | 1.51 | 1.65 | 3.70 | 1.41 |  | 2.56 | 2.64 | 4.39 | 3.60 | 2.92 |
| ATT9 | 2.85 | 2.10 | 3.43 | 4.35 | 3.12 | 1.49 | 1.79 | 3.15 |  |  | 2.27 | 2.20 | 4.43 | 3.60 | 2.68 |
| ATT10 | 2.35 | 2.46 | 3.57 | 4.33 | 3.08 | 1.44 |  | 2.59 | 1.99 | 1.58 | 2.04 | 2.12 | 3.20 | 3.20 | 2.48 |
| ATT11 | 2.77 | 4.06 | 4.03 |  | 3.87 | 1.71 | 2.44 | 3.24 | 2.30 | 2.14 | 2.48 | 3.33 | 4.17 |  | 3.38 |
| ATT12 | 2.33 | 3.35 | 3.68 | 4.68 | 3.65 | 1.41 | 2.15 | 2.38 | 2.00 | 1.90 | 2.03 | 3.05 | 2.90 | 3.39 | 3.05 |
| ATT13 |  |  | 1.80 | 1.90 | 1.53 |  |  |  |  |  |  | 1.96 | 1.79 | 1.74 | 1.62 |
| ATT14 |  | 1.88 | 1.95 | 2.89 | 1.98 |  |  | 1.62 |  |  | 1.64 | 2.06 | 1.98 | 2.39 | 1.90 |
| ATT15 | 1.39 |  |  | 1.91 |  |  |  |  |  |  | 1.52 |  |  | 1.70 | 1.39 |
| PL1 | 1.41 | 2.55 | 2.36 | 1.84 | 1.81 |  | 2.82 | 2.56 | 1.49 | 2.40 | 1.41 | 3.85 | 2.46 | 1.66 | 2.27 |
| PL2 | 1.76 |  | 3.11 | 2.20 |  |  |  | 3.31 | 1.66 | 2.72 | 1.69 | 3.84 | 3.01 | 1.91 | 2.66 |
| PL3 | 1.94 | 2.37 | 2.10 | 2.10 | 1.84 |  | 3.15 | 3.27 | 1.32 | 2.65 | 1.70 | 3.40 | 2.41 | 1.67 | 2.47 |
| PL4 |  | 1.22 | 1.22 |  | 1.25 | 1.00 | 2.26 | 2.23 |  | 1.49 |  | 1.92 | 1.88 |  | 1.37 |
| PL5 |  |  |  | 1.24 | 1.29 |  | 2.03 | 2.45 |  | 1.57 |  | 1.78 | 2.10 |  | 1.44 |
| PBC1 | 4.01 | 1.93 | 2.69 | 4.42 | 2.94 | 1.45 | 1.80 | 2.42 | 2.34 | 1.69 | 2.99 | 2.12 | 3.11 | 3.61 | 2.74 |
| PBC2 | 4.33 | 2.05 | 2.90 | 4.60 | 3.11 | 1.68 | 2.05 | 3.37 | 2.75 | 2.06 | 3.32 | 2.66 | 3.33 | 4.06 | 3.16 |
| PBC3 | 1.36 | 1.76 |  | 1.26 | 1.27 |  | 2.24 |  | 1.40 |  | 1.36 | 2.50 | 1.34 | 1.32 | 1.70 |
| PBC4 | 1.91 | 1.57 | 1.83 | 1.37 | 1.50 | 1.41 |  | 2.35 |  | 1.25 | 2.01 | 1.51 | 2.00 | 1.38 | 1.61 |
| PBC5 | 1.51 | 1.87 |  |  |  |  | 1.57 |  | 1.24 |  |  | 1.97 |  |  | 1.38 |
| PBC6 | 3.13 | 1.61 | 1.65 | 1.57 | 1.51 | 1.45 |  | 1.72 |  |  | 1.66 | 1.70 | 1.58 | 1.48 | 1.58 |
| PBC7 | 3.00 | 2.29 | 2.03 | 2.34 | 2.32 | 1.72 | 1.41 | 1.75 |  | 1.35 | 2.30 | 2.19 | 1.97 | 1.98 | 2.12 |
| PBC8 |  | 2.35 | 1.82 | 2.73 | 2.35 | 1.81 | 1.61 | 2.53 | 1.82 | 1.75 | 2.72 | 2.20 | 2.14 | 2.79 | 2.47 |
| SN1 | 1.65 | 1.88 |  | 2.31 | 1.73 | 1.46 | 1.98 |  | 1.37 | 1.44 | 1.64 | 1.97 | 1.24 | 2.15 | 1.79 |
| SN2 | 1.74 | 1.71 |  | 1.39 | 1.50 | 1.58 | 1.58 |  |  |  | 1.69 | 1.78 |  | 1.51 | 1.64 |
| SN3 | 2.25 | 2.06 | 2.28 | 2.44 | 2.29 | 1.60 | 1.68 | 1.48 | 2.47 | 1.61 | 1.99 | 2.00 | 2.19 | 3.29 | 2.27 |
| SN4 | 2.38 | 2.12 | 2.27 |  | 2.83 |  | 2.31 | 2.06 | 1.93 | 1.86 | 1.92 | 2.42 | 2.49 | 4.11 | 2.68 |
| SN5 | 2.62 | 1.98 |  | 2.80 | 2.27 | 1.77 | 2.10 | 1.83 |  | 1.65 | 2.04 | 2.29 | 1.69 | 2.47 | 2.19 |
| SN6 | 2.52 | 1.97 | 1.37 | 2.79 | 2.05 | 1.54 | 2.09 | 1.59 | 1.13 | 1.59 | 2.07 | 2.16 | 1.35 | 2.13 | 2.06 |
| SN7 | 1.69 | 1.68 | 1.44 | 1.59 | 1.53 | 2.31 | 1.61 | 1.60 | 1.68 | 1.53 | 1.97 | 1.73 |  | 1.60 | 1.69 |
| INT1 |  |  |  |  |  |  | 1.98 |  |  |  |  | 1.40 | 1.58 |  |  |
| INT2 |  | 1.50 | 3.27 |  | 1.15 | 1.57 | 2.57 | 2.89 | 1.85 | 2.05 |  | 2.18 | 3.23 |  | 1.95 |
| INT3 |  | 1.62 | 3.18 |  |  | 1.82 | 2.53 | 2.06 | 1.88 | 2.09 |  | 2.19 | 2.72 |  | 1.80 |
| INT4 | 2.39 | 1.38 |  | 1.45 | 1.30 |  |  |  |  |  |  |  |  |  |  |
| INT5 | 3.33 | 2.68 | 2.51 | 2.99 | 2.60 |  |  |  |  |  |  |  |  |  |  |
| INT6 | 1.53 | 2.23 | 2.62 | 3.11 | 2.40 |  |  |  |  |  |  |  |  |  |  |
| INT7 | 1.58 | 2.27 | 1.41 | 1.71 | 1.60 | 1.57 | 2.25 | 1.69 | 1.35 | 1.64 | 1.63 | 2.31 | 1.89 | 1.47 | 1.84 |
| INT8 |  | 1.94 |  | 1.92 |  | 1.34 | 1.61 | 1.39 | 1.17 | 1.34 | 1.63 | 1.95 | 1.64 | 1.47 | 1.69 |
| ATT -> INT | 1.43 | 1.82 | 1.41 | 3.23 | 1.86 | 1.41 | 1.32 | 1.27 | 1.48 | 1.30 | 1.86 | 2.03 | 1.72 | 2.84 | 2.09 |
| PBC -> INT | 1.20 | 1.42 | 1.38 | 2.13 | 1.54 | 1.79 | 1.46 | 1.15 | 1.24 | 1.35 | 1.60 | 1.84 | 1.38 | 2.07 | 1.76 |
| PL -> INT | 1.00 | 1.06 | 1.03 | 1.89 | 1.07 | 1.14 | 1.03 | 1.25 | 1.09 | 1.02 | 1.03 | 1.07 | 1.07 | 1.32 | 1.01 |
| SN -> INT | 1.33 | 2.03 | 1.36 | 3.39 | 1.92 | 1.83 | 1.46 | 1.49 | 1.64 | 1.46 | 1.67 | 2.01 | 1.99 | 3.16 | 2.25 |
| INT -> ABEH |  |  |  |  |  |  |  |  |  |  | 1.49 | 1.56 | 1.14 | 1.39 | 1.43 |
| PBC -> ABEH |  |  |  |  |  |  |  |  |  |  | 1.49 | 1.56 | 1.14 | 1.39 | 1.43 |

**Table A.5.** Multigroup/country analysis

|  | Kenya - Uganda | P value | Kenya - Tanzania | P value | Kenya - Rwanda | P value | Uganda - Tanzania | P value | Uganda - Rwanda | P value | Tanzania - Rwanda | P value |
| --- | --- | --- | --- | --- | --- | --- | --- | --- | --- | --- | --- | --- |
| **Non-users** | | | | | | | | | | | | |
| ATT -> INT | 0.006 | 0.466 | -0.411 | 0.019 | -0.352 | 0.043 | -0.417 | 0.003 | -0.358 | 0.015 | 0.059 | 0.349 |
| PBC -> INT | 0.040 | 0.376 | 0.232 | 0.077 | 0.169 | 0.111 | 0.192 | 0.098 | 0.128 | 0.143 | -0.063 | 0.347 |
| PL -> INT | 0.159 | 0.232 | 0.241 | 0.152 | 0.320 | 0.038 | 0.081 | 0.275 | 0.161 | 0.070 | 0.080 | 0.351 |
| SN -> INT | -0.151 | 0.166 | 0.185 | 0.098 | 0.114 | 0.243 | 0.336 | 0.016 | 0.265 | 0.064 | -0.071 | 0.326 |
| **Users** | | | | | | | | | | | | |
| ATT -> INT | -0.027 | 0.412 | -0.138 | 0.115 | 0.072 | 0.251 | -0.110 | 0.209 | 0.100 | 0.221 | 0.210 | 0.045 |
| PBC -> INT | 0.257 | 0.037 | 0.137 | 0.121 | 0.163 | 0.108 | -0.121 | 0.193 | -0.094 | 0.269 | 0.027 | 0.414 |
| PL -> INT | -0.024 | 0.459 | 0.318 | 0.008 | -0.144 | 0.158 | 0.342 | 0.010 | -0.120 | 0.201 | -0.462 | 0.000 |
| SN -> INT | -0.155 | 0.150 | 0.208 | 0.074 | -0.189 | 0.107 | 0.363 | 0.018 | -0.034 | 0.421 | -0.397 | 0.009 |
| **Pooled sample** | | | | | | | | | | | | |
| **Total effects** | | | | | | | | | | | | |
| ATT -> INT | -0.221 | 0.034 | -0.504 | 0.000 | -0.168 | 0.082 | -0.282 | 0.009 | 0.053 | 0.344 | 0.335 | 0.002 |
| INT -> ABEH | -0.017 | 0.357 | -0.048 | 0.139 | -0.023 | 0.314 | -0.031 | 0.230 | -0.006 | 0.451 | 0.025 | 0.273 |
| PBC -> ABEH | 0.074 | 0.028 | 0.053 | 0.074 | 0.001 | 0.493 | -0.021 | 0.301 | -0.074 | 0.031 | -0.053 | 0.079 |
| PBC -> INT | 0.237 | 0.008 | 0.267 | 0.002 | 0.123 | 0.109 | 0.030 | 0.382 | -0.114 | 0.133 | -0.145 | 0.071 |
| PL -> INT | 0.144 | 0.088 | 0.245 | 0.004 | 0.052 | 0.281 | 0.101 | 0.129 | -0.093 | 0.117 | -0.193 | 0.002 |
| SN -> INT | 0.027 | 0.416 | 0.373 | 0.000 | -0.017 | 0.441 | 0.347 | 0.004 | -0.044 | 0.382 | -0.391 | 0.001 |
| **Indirect effects** | | | | | | | | | | | | |
| ATT -> INT -> ABEH | -0.018 | 0.048 | -0.056 | 0.001 | -0.015 | 0.130 | -0.039 | 0.020 | 0.003 | 0.396 | 0.042 | 0.027 |
| PBC -> INT -> ABEH | 0.013 | 0.180 | 0.013 | 0.186 | 0.003 | 0.435 | 0.000 | 0.500 | -0.010 | 0.180 | -0.010 | 0.191 |
| PL -> INT -> ABEH | 0.011 | 0.084 | 0.025 | 0.004 | 0.004 | 0.281 | 0.014 | 0.088 | -0.007 | 0.138 | -0.022 | 0.006 |
| SN -> INT -> ABEH | -0.006 | 0.411 | 0.019 | 0.146 | -0.012 | 0.288 | 0.025 | 0.092 | -0.006 | 0.381 | -0.031 | 0.032 |

**Appendix B: Structural models with path coefficients, figures in brackets are p-values**

1. **Structural models for PPT non-users**


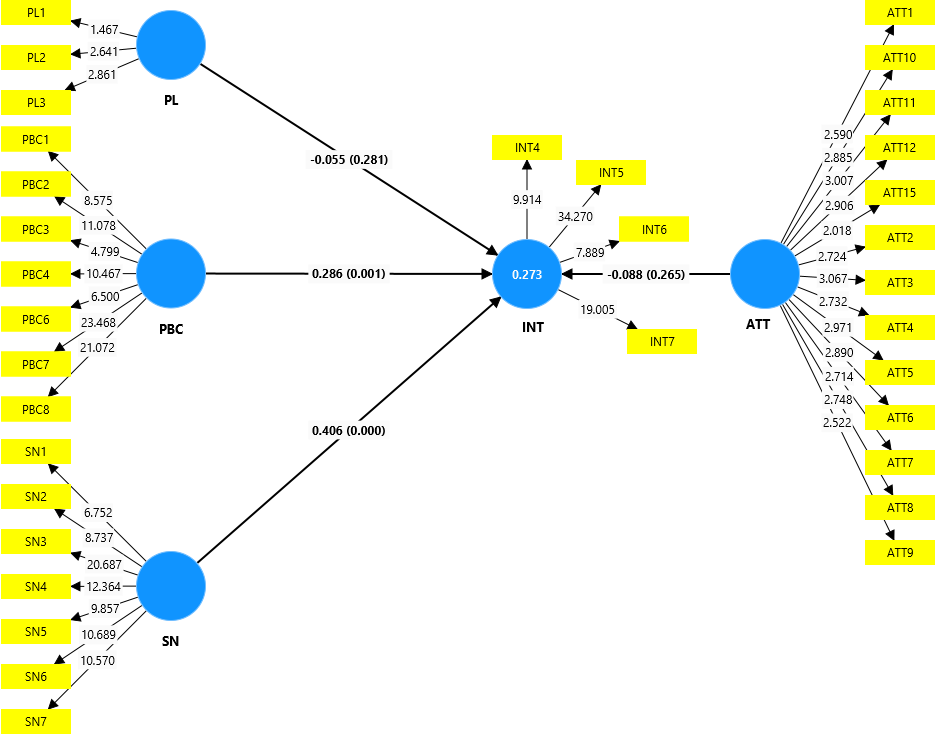


**Figure B.1.** Structural model for Kenya non-users


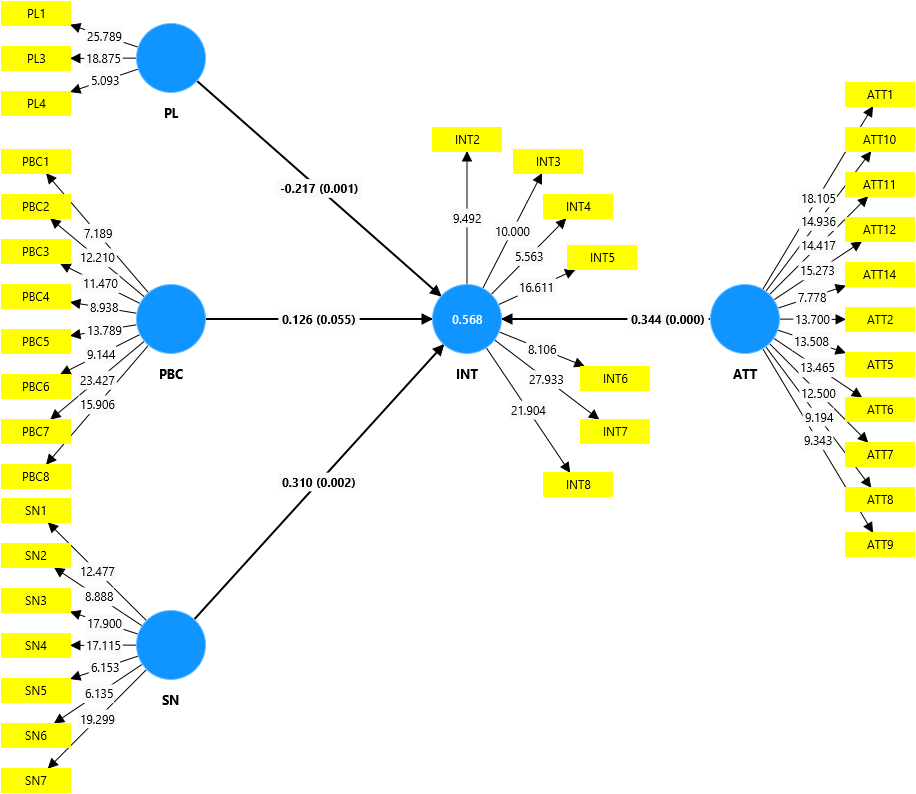


**Figure B.2.** Structural model for Rwanda non-users


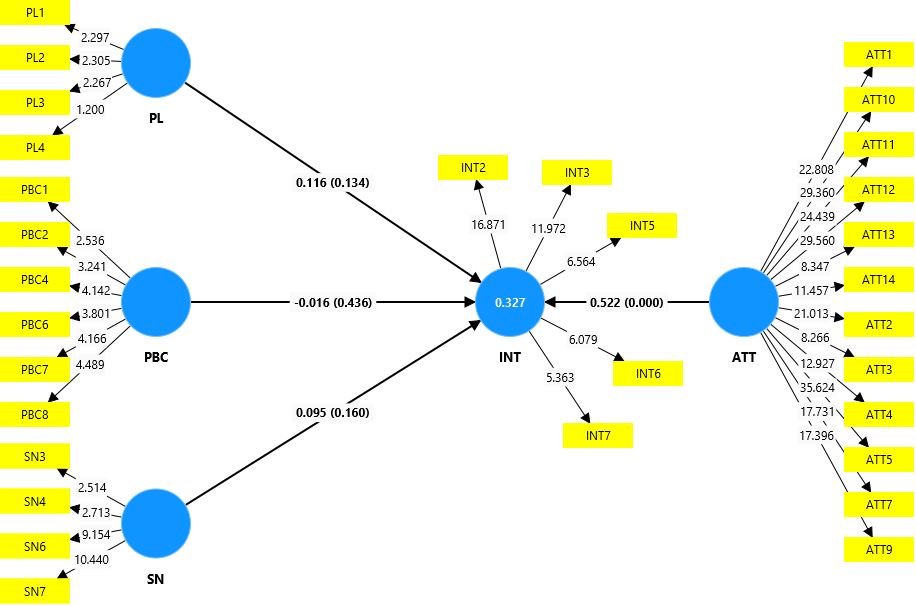


**Figure B.3.** Structural model for Tanzania non-users


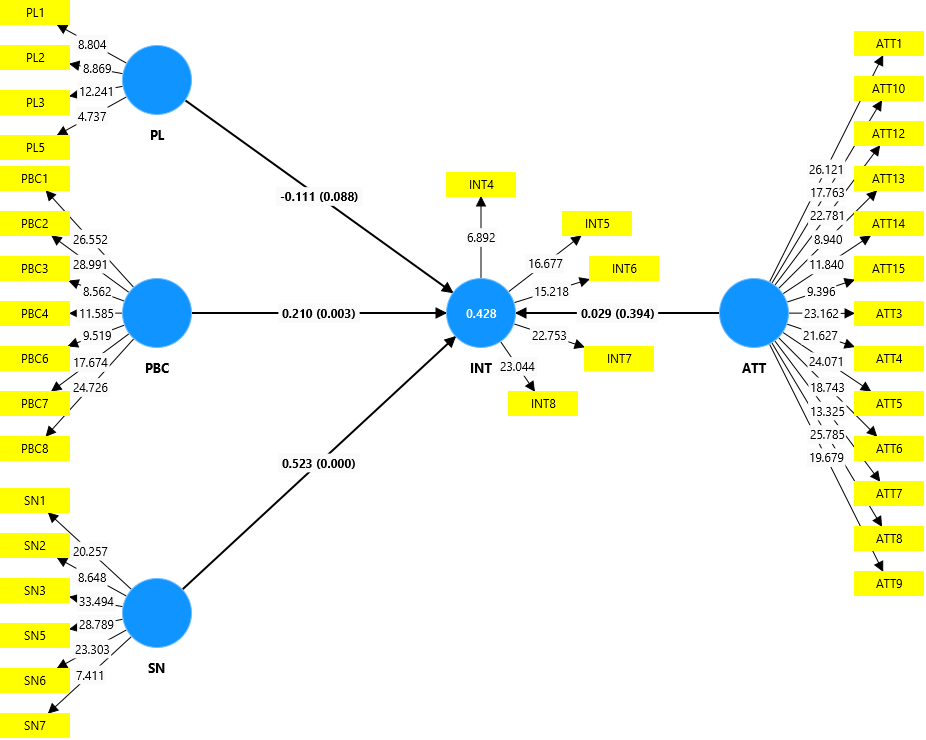


**Figure B.4.** Structural model for Uganda non-users

1. **Structural models for PPT users with path coefficients**


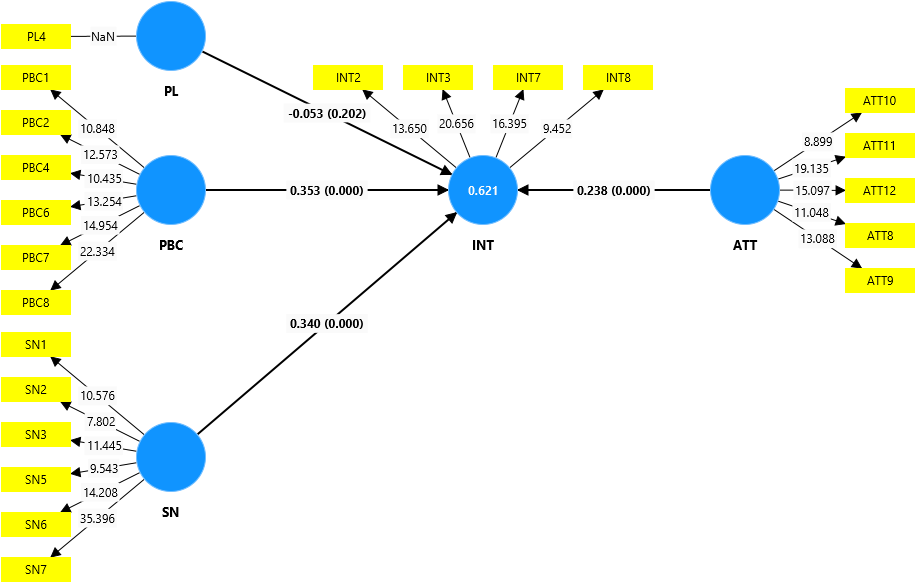


**Figure B.5.** Structural model for Kenya PPT users


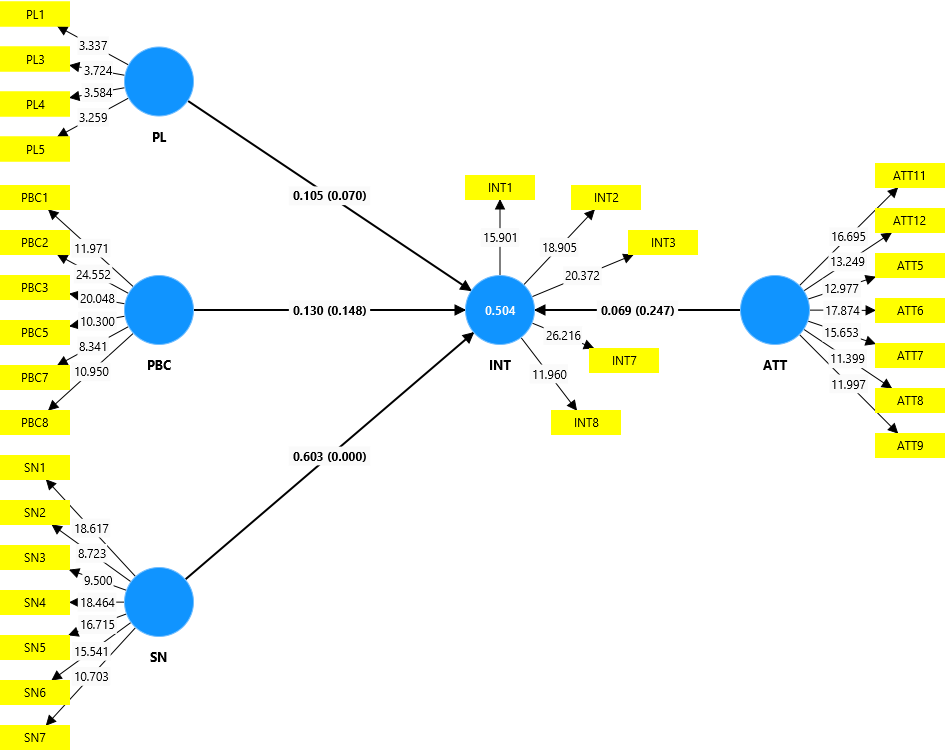


**Figure B.6.** Structural model for Rwanda PPT users


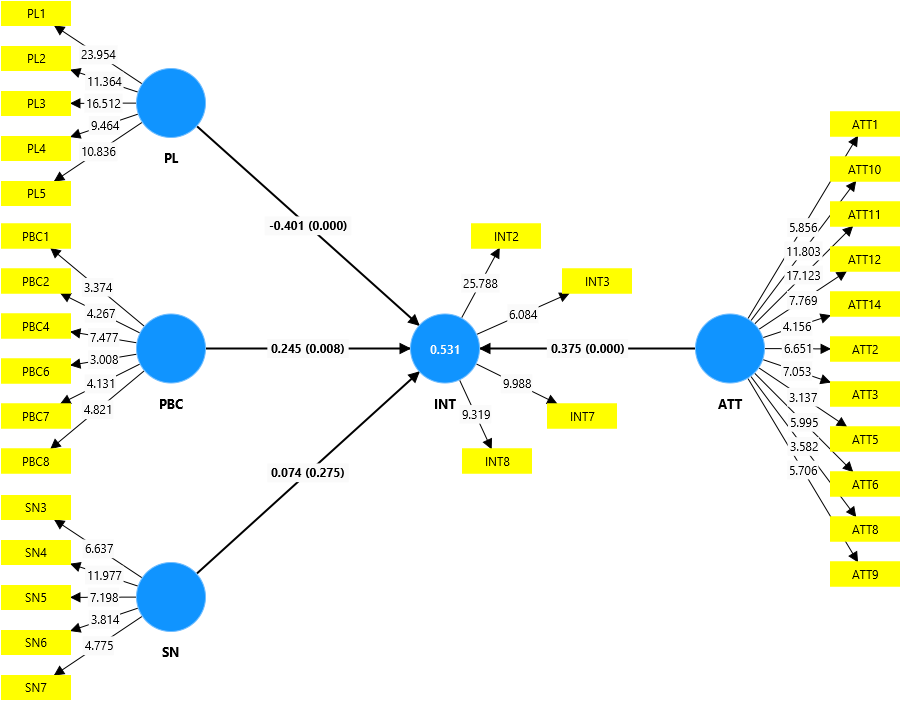


**Figure B.7.** Structural model for Tanzania PPT users


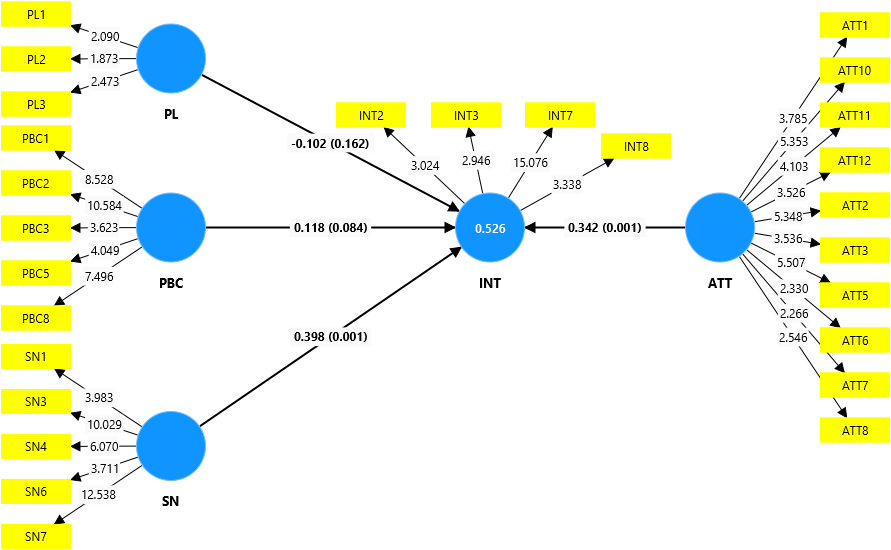


**Figure B.8.** Structural model for Uganda PPT users

1. **Structural models for the pooled samples with path coefficients**


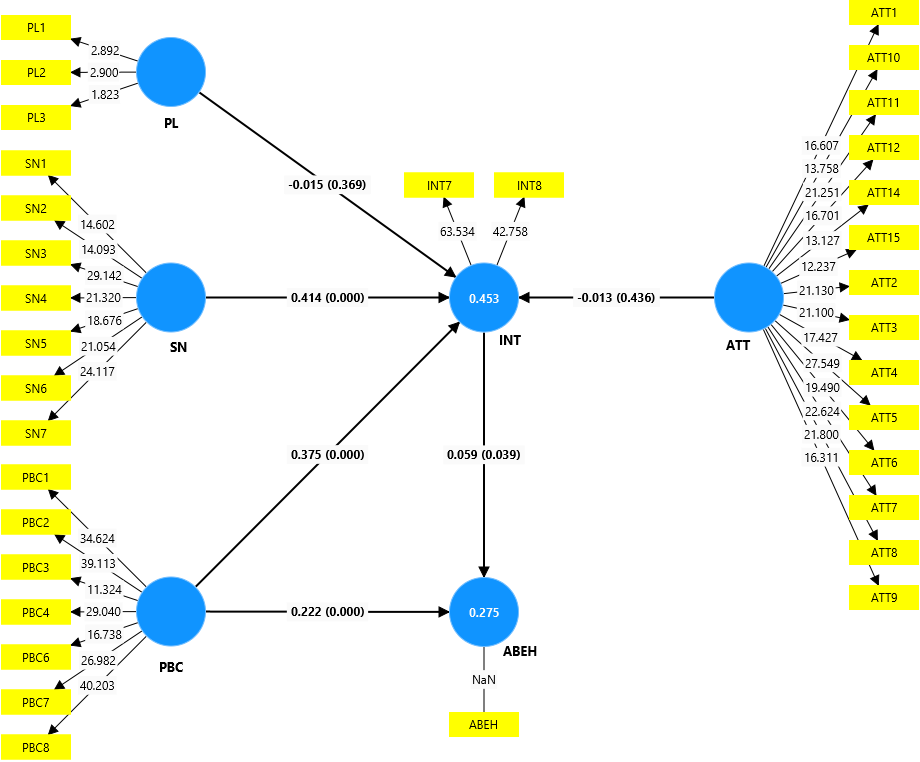


**Figure B.9.** Structural model for the Kenya pooled sample


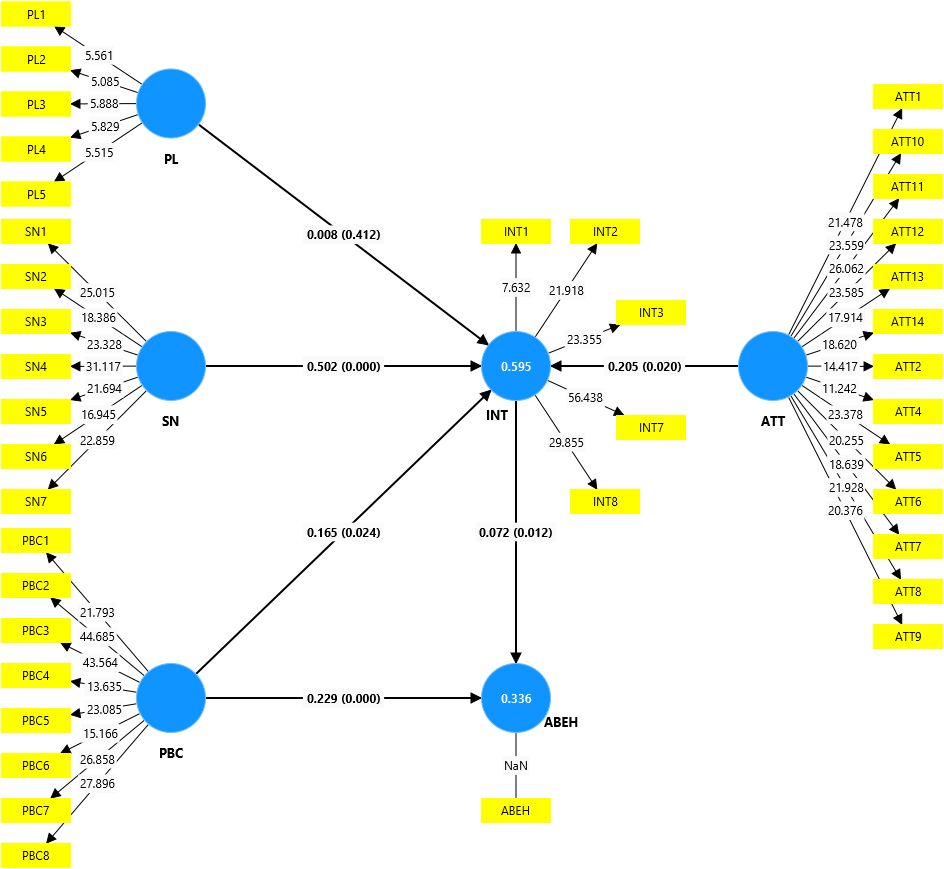


**Figure B.10.** Structural model for the Rwanda pooled sample


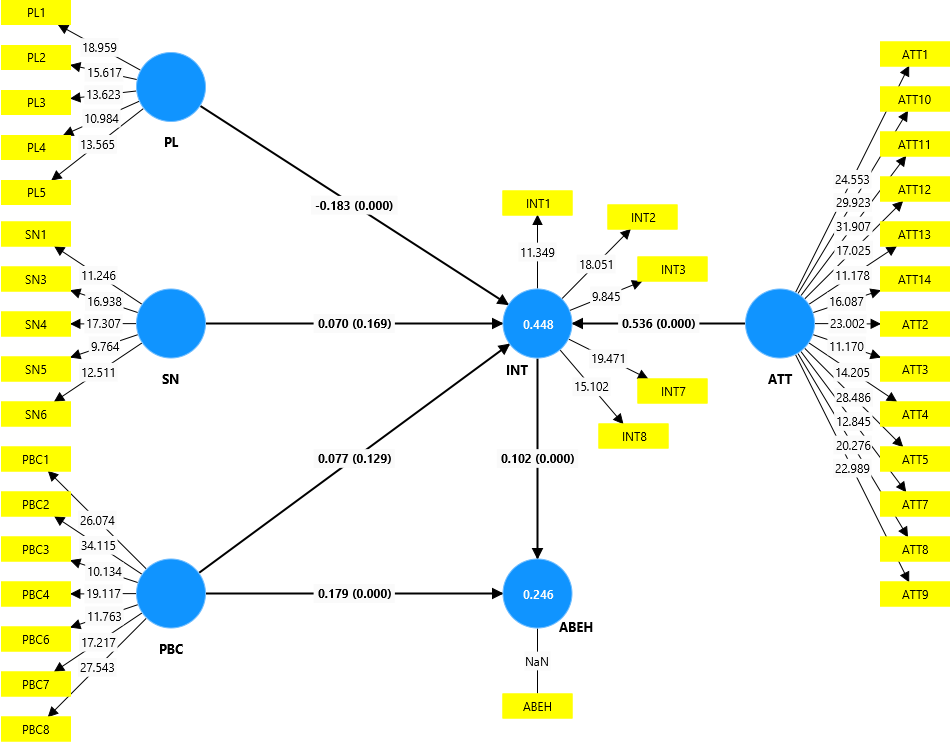


**Figure B.11.** Structural model for the Tanzania pooled sample


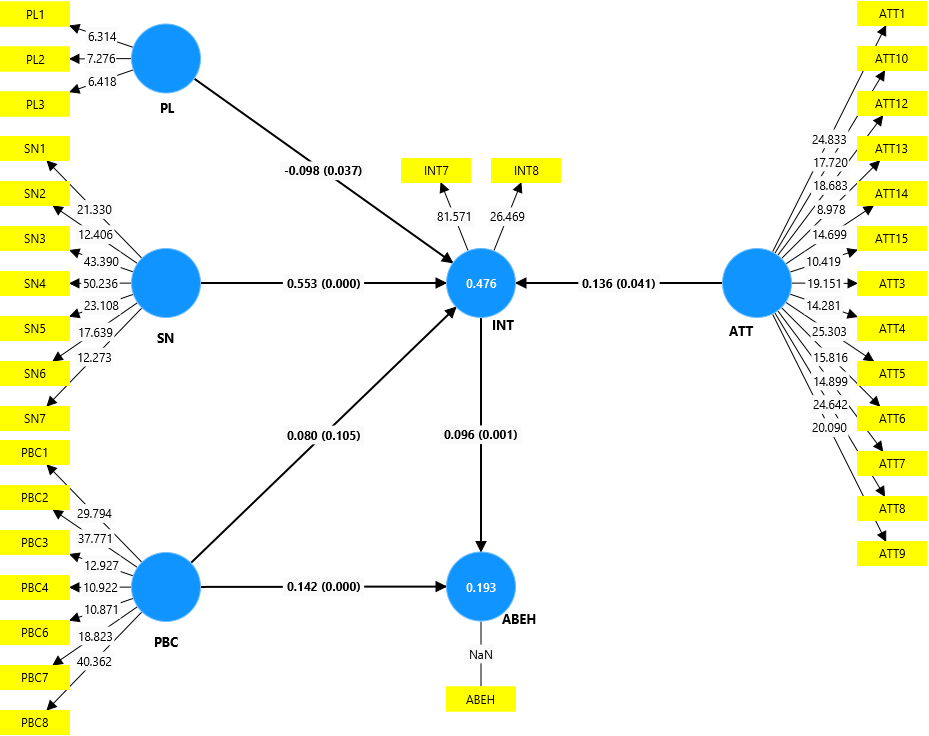


**Figure B.12.** Structural model for the Uganda pooled sample
